# The influence of task difficulty, social tolerance and model success on social learning in Barbary macaques

**DOI:** 10.1038/s41598-022-26699-6

**Published:** 2023-01-20

**Authors:** Ivan Garcia-Nisa, Cara Evans, Rachel L. Kendal

**Affiliations:** Durham Cultural Evolution Research Centre, Department of Anthropology, Durham University, Durham, UK

**Keywords:** Cultural evolution, Social evolution, Behavioural ecology, Evolutionary ecology, Biological anthropology, Animal behaviour

## Abstract

Despite playing a pivotal role in the inception of animal culture studies, macaque social learning is surprisingly understudied. Social learning is important to survival and influenced by dominance and affiliation in social animals. Individuals generally rely on social learning when individual learning is costly, and selectively use social learning strategies influencing what is learned and from whom. Here, we combined social learning experiments, using extractive foraging tasks, with network-based diffusion analysis (using various social relationships) to investigate the transmission of social information in free-ranging Barbary macaques. We also investigated the influence of task difficulty on reliance on social information and evidence for social learning strategies. Social learning was detected for the most difficult tasks only, with huddling relations outside task introductions, and observation networks during task introductions, predicting social transmission. For the most difficult task only, individuals appeared to employ a social learning strategy of copying the most successful demonstrator observed. Results indicate that high social tolerance represents social learning opportunities and influences social learning processes. The reliance of Barbary macaques on social learning, and cues of model-success supports the costly information hypothesis. Our study provides more statistical evidence to the previous claims indicative of culture in macaques.

## Introduction

Social learning is defined as ‘learning influenced by the observation of, or interaction (e.g., vocal signals) with, a conspecific, or its products’ (e.g., scent marks)^1,2^. The transmission of social information, via social learning, within a group is required for the maintenance of animal traditions, or culture, and can be important to survival^3^. Social learning allows naïve individuals to acquire information relevant to many life skills or adaptive behaviours invented by knowledgeable conspecifics^3^. For instance, the social transmission of foraging techniques helps many primate species make use of a wide diversity of food resources^4–6^.

The acquisition of knowledge from others may benefit individuals’ fitness because it allows them to acquire adaptive information while minimizing the costs (e.g. time and energy invested in searching for food, predation risk) of learning by themselves^7–10^. However, information obtained by social learning may be maladaptive (e.g., if socially acquired behaviours are maintained even when the environment changes)^7,8,11^. Accordingly, individuals face a trade-off between costly but accurate and reliable information obtained by trial-and-error (asocial learning) and potentially unreliable but safe and easy-to-obtain social information^7,12^. Theoretical models of the evolution of social learning suggest that individuals must often engage in selective social learning influencing whether they learn for themselves and if not, what and from whom they learn socially^7,8,11,13,14^. Such social learning strategies^14,15^ include copying frequent behaviours (e.g., copy the majority), ‘whom’ to copy (e.g., copying the most prestigious or skilled individual), ‘when’ to copy (e.g., copy when uncertain) and ‘what’ to copy (e.g., copy the variant with the highest payoff).

One method to test for social learning consists of using open-diffusion experiments in which extractive foraging tasks with two possible actions to access rewards are presented^16–18^. The two-action paradigm allows testing for socially influenced option/action preferences at the individual or group level^16^. Open-diffusion experiments allow testing for the spread of novel behaviours within the ecologically valid context of the social group^16,18^. As nonhumans rely on social learning only when asocial learning is challenging^10,19^ the experimental tasks involved must be appropriately difficult, as demonstrated in primates^10,20^. Open-diffusion studies also enable the relationship between social dynamics and social learning to be investigated^21^. Social relations are influenced by the levels of social tolerance towards others in proximity, so that those that spend more time together, are more likely to learn from each other^18,22^.

Network-based diffusion analysis (NBDA)^23^ is a statistical method to test for the social transmission of information in groups of animals. The method has been extended^24–26^ and can now be based on the order or time in which individuals acquire a novel trait (OADA—order of acquisition diffusion analysis, TADA—time of acquisition diffusion analysis). The analysis assumes that the transmission of a novel trait will be faster between individuals that have strong social connections. NBDA compares the order or time of acquisition of the trait to the pattern of connections in a group’s social network^22,23^. The analysis also allows other factors that may influence social and asocial learning rates to be controlled, such as sex, age, and social rank^24,25^.

In nonhuman primates, a growing number of studies have provided evidence of social transmission using NBDA, tracking the spread of social information through networks based on (i) affiliative interactions (wild red-fronted lemurs, *Eulemur rufifrons*, Ref.^27^; grooming and co-feeding in wild bearded capuchins, *Sapajus libidinosus*, Ref.^28^), (ii) associations (captive squirrel monkeys, *Saimiri sciureus*, Ref.^29^; captive bonobos, *Pan paniscus*, Ref.^30^), (iii) rank similarity (wild ring-tailed lemurs, *Lemur catta*, Ref.^20^) or (iv) who observes whom during the spread of innovative behaviours or task introductions (wild chimpanzees, *Pan troglodytes schweinfurthii*, Ref.^5^; wild vervet monkeys, *Chlorocebus pygerythrus*, Ref.^31^). However, macaque species are conspicuously absent in these recent studies of social learning. This is surprising given that it was the innovation and spread of sweet potato-washing in a group of provisioned Japanese macaques^32^ that initiated use of the term ‘animal pre-culture’ and discussions about the possibility of non-human behavioural (cultural) traditions, back in the early 1950s^33^.

Some studies have reported observations of social transmission and maintenance of novel behaviours in macaques, especially in tool-using species^33–38^. However, these observations are often conflicting, probably due to use of different methodological approaches and the lack of powerful standard tests at the time^33–36,39^. Social learning has been reported for the transmission of feeding techniques in Tonkean macaques (*Macaca tonkeana*, Ref.^34^), abnormal behaviours of captive rhesus macaques (*Macaca mulatta*, Ref.^40^) and tool-use in long-tailed (*Macaca fascicularis*, Refs.^41–43^; but see^44^) and Japanese macaques (*Macaca fuscata*, Refs.^33,45^). Moreover, individuals of different macaque species have been observed copying the behaviour of (i) genetically-related conspecifics (Japanese macaques, Refs.^46,47^; long-tailed macaques, Ref.^43^), (ii) strong affiliates or (iii) the most productive and proficient tool-users (long-tailed macaques, Ref.^43^). In addition, cases of Japanese macaques copying the behavioural variant of the neighbouring group have been reported^45,48^. However, these few studies did not directly test for the transmission of social information. Here, we use NBDA to test directly for social learning throughout a macaque group^24,26^.

We investigated social learning in a large semi-free ranging group of Barbary macaques. They are considered a tolerant species within macaques due to high levels of affiliative displays^49–54^, and thus ideal for application of NBDA to investigate the role of social tolerance in social learning. Recent research has shown that Barbary macaques are capable of innovation (a pre-requisite for behavioural traditions) and cooperation (requiring high levels of social tolerance, important for social transmission of information) in novel foraging and problem-solving contexts^53,55^. The main evidence for social learning in Barbary macaques comes from studies on vocal development and communication. Fischer (2004) suggested that despite the production of vocalizations in Barbary macaques likely being not learned, call comprehension may be based on learning influenced by exposure to others’ vocalizations^56^. Moreover, the gradual development observed in the emergence of individual vocal recognition among Barbary macaques also suggests a social influence in the acquisition of call comprehension abilities^57^. However, the only study, we are aware of, that investigated social learning of a novel foraging task in Barbary macaques, failed to find evidence for social learning (social facilitation)^55^. We tested three foraging tasks of increasing difficulty and used NBDA to track the spread of information through social networks depicting different levels of social tolerance in different contexts: (a) kinship as a measure of tolerance due to genetic similarity, (b) affiliative interactions (grooming, huddling) and associations (proximity within 1 and 5 m) outside task introductions as measures of tolerance due to frequent affiliation/time spent together, each representing different aspects of affiliative relationships according to previous findings^58^, (c) observation networks (within 1 and 5 m) during task introduction times as measures of different levels of social tolerance during highly competitive contexts.

We expected to find evidence of social learning in Barbary macaques as it is observed in other macaque species. Moreover, Barbary macaques display many characteristics that are useful for social learning and problem-solving. Specifically, since affiliative relations and observation networks are said to represent social learning opportunities^22,59^, we predicted that (1a) kinship networks, (1b) networks based on affiliative interactions and associations, and (1c) observation networks during task introductions, will predict the patterns of social information transmission. We also expected to find evidence of social learning strategies^14^. Specifically, as primates are more likely to employ social learning when asocial learning is challenging^12^ we predicted (2a) that we will find more evidence for social learning as task difficulty increased. Accordingly, we predicted that kinship, affiliative and observation networks will predict the patterns of social transmission more strongly as task difficulty increased. Finally, we expected to find evidence of context-dependent social learning strategies for tasks where social learning is required, as indicated by studies of many primate species, including macaques^48,60–62^. We predicted (2b) that we may find evidence for ‘who’ strategies: (i.e. affiliation and proficiency bias such as copying strong affiliates or the most successful demonstrator at the task, respectively, Ref.^43^), and ‘what’ strategies (i.e. payoff-bias such as copying the task option that yields the highest rewards, Ref.^63^) in the Barbary macaques.

## Methods

### Statement of ethical approval

Barbary macaques at Trentham Monkey Forest were well habituated to human presence. Thus, our observation of them for collection of social network data had no ethical implications. Potential ethical issues encountered during pilot tests of the extractive foraging tasks were solved and reported to the ethics committee for approval of the research project. All procedures were approved by the Animal Welfare Ethical Review Board (AWERB) of Durham University. The study was entirely carried out in accordance with the ethical guidelines of the Association for the Study of Animal Behaviour (ASAB).

### Study site and subjects

Data collection took place with a group of free-ranging Barbary macaques in Trentham Monkey Forest (England, United Kingdom), a 60-acre forest that can be visited by members of the public walking along a ¾ mile pathway. Monkeys received daily feeds of fruit, vegetables, nuts and seeds to supplement natural resources. The park housed two groups, each having separate minimally overlapping home ranges and containing 8–10 matrilines. We studied the ‘German group’ (N = 61), consisting of 22 adult males, 27 adult females, 5 sub-adult males, 2 sub-adult females and 5 infants. During the study, one adult male died and 7 infants were born, with 4 surviving. Due to unreliable identification, subjects younger than 3 years (all infants, Ref.^64^) were excluded from the study (resulting in N = 56). All experimental apparatuses were previously piloted in the ‘French group’ (N = 75).

### Social networks

Behavioural data were collected from June to September 2011 (76 days, 134.5 h; 115–175 (median = 145) minutes per subject) to create various social networks. Data regarding three socio-positive behaviours (grooming, huddling and proximity, see Table 1 for definitions) were collected outside of task introduction times. During 5 min focal follows, all instances of grooming and huddling involving the focal subject were recorded, whilst individuals within 1 m and 5 m of the focal (representing different levels of tolerance) were recorded using scan samples at 0 and 4 min. Additionally, agonistic and submissive interactions (Table 1) between any visible subjects were collected on an all-occurrence basis to determine social ranks (Supplementary Information S2). Simple Ratio Index (SRI; Ref.^65^) was used to calculate the strength of relations between pairs of individuals (edge weight) for all socio-positive behavioural networks.

**Table 1:** Ethogram of socio-positive, agonistic and task-related behaviours of a group of Barbary macaques (*Macaca sylvanus*) at Trentham Monkey Forest (UK).

Behaviour		Description
Affiliative interactions	Grooming	When individuals manipulate another’s fur using hands or mouth. A new bout was considered when groomer and groomee exchanged roles
	Huddling	Two or more individuals are resting (i.e. to be still, sitting or lying, asleep or awake, with the eyes closed and/or facing down) with bodies in direct contact, lateral or ventral. Arms may be wrapped around one another
Associations	Proximity	Two or more stationary individuals are found within 1 m and between 1-5 m. As moving individuals may have little control over whom they are close to, these events were not considered as proximity
Agonistic displays	Threaten	Rounded mouth or open mouth bared-teeth display with eyelids raised. May include vocalisations (pants, barks, noisy and complex screams) and a tense body posture (front body lowered) and/or a small movement (lunge) toward the monkey being threatened (sometimes hitting the ground with the hand)
	Re-directing aggression	When subjects threaten third-parties while being threatened by other individuals
	Chase	When an individual runs towards another, who is moving rapidly away, displaying threatening behaviours. Involves quick movements with full body
	Physical assault	Physical contact, in which a monkey pushes with hands, hits or bites a conspecific. It also includes rough and tumble fights. The types of aggression (e.g. push, bite, hit) as well as injuries incurred were noted
Submissive behaviours	Move away	When threatened and/or chased, the target of the agonistic interaction moves away from the aggressor. It also includes events when an individual moves away from a place, a food patch or a partner that is being approached (within 1 m) by another conspecific
	Submissive grin	Facial expression with retracted lips usually produced in response to a threat, normally accompanied by teeth-chattering and presentation of the rear to the aggressor
	Scream or cry	High-pitched vocalization usually issued in response to a threat and that may, or may not, elicit response from coalition partners
Absence	Not visible	When the subject, without leaving the outside enclosure, goes out of sight of the researcher (e.g. behind an obstacle) (BDG and TG)
Task-related behaviours	Bout	A bout started when an individual approached within 0.5 m of the task and ended when the individual moved further than 0.5 m of the task
	Task contact	Exploratory behaviours involving inspecting, touching, biting, leaning-on and pulling the task that do not involve solving the actions and retrieving rewards from the task
	Unsuccessful manipulation	A monkey manipulates the moving parts of the tasks (e.g. the door or rotating disc) and/or places hand(s) into the retrieving hole but does not retrieve raisins from the task
	Successful manipulation	A manipulation of the task that results in raisins being retrieved from inside the task
	Displaced from task	Individual at task moves away when a nearby individual directs agonistic behaviours towards them (i.e. face threat, chase off, hit, bite) or another individual approaches the task (an approach being within 0.5 m of the task)
	Leaves task	Animal who is at task moves back or away (animal's whole body is beyond 0.5 m) from it of its own accord (i.e. without being displaced, or the session ending or task being broken/refilled). If the animal moves away to threaten or chase off another individual who is in proximity or approaching but then returns immediately back to the task, the animal was not recorded as leaving the task
	Observing	Individuals within 5 m of the task attend (i.e. head and/or gaze oriented towards) the individual at task (i.e. within 0.5 m of the task) or manipulating the task. Distance from task of observers was noted as within 1 m or between 1-5 m
	Refill	Researcher approaches task (0.5 m) and inserts rewards inside. If individuals were able to observe the refill, their identity and distance from the task was noted

Observation networks were based on who observed whom (within 1 m and 5 m) interacting with the introduced tasks during each interaction bout (Table 1). The number of such task interaction bouts was not sufficient to obtain accurate measures of the strength of relations for observation networks. Instead, the connections between individuals in observation networks are directed from the observer to the demonstrator and represent that, at least, one observation event was recorded between those individuals. Accordingly, in observation networks, values for each pair of individuals could only be 0 or 1, where 0 indicated that the observer never observed the demonstrator solving the task, and 1 indicated that the observer observed one or more successful task manipulations from the demonstrator. Kinship networks were built using the coefficient of mother relatedness (0.5 × degree of relationship), as indicated by park records, as edge weights.

### Description of the tasks

**Figure 1:**
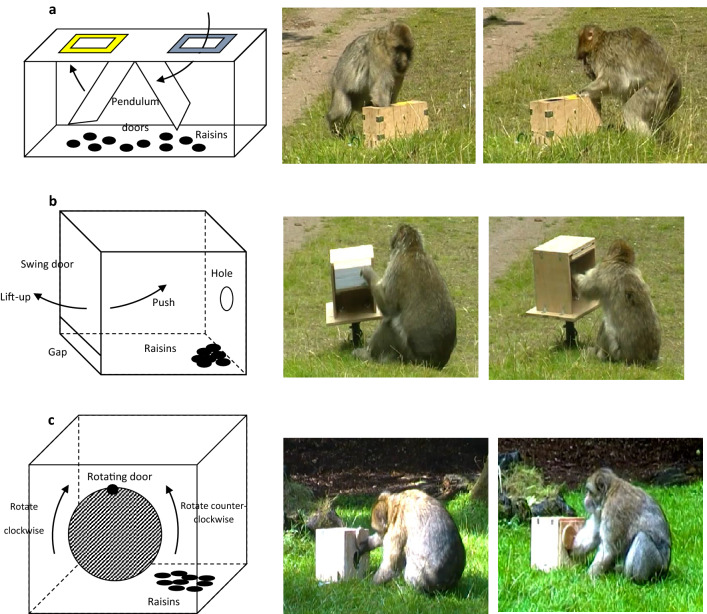
Diagrams (left) and photos (right) of the foraging tasks presented to a group of Barbary macaques (Macaca sylvanus) at Trentham Monkey Forest (UK). (**a**) Illustration of the blue/yellow task being used by a mid-ranking adult female (FO) retrieving rewards from the blue hole (left) and a low-ranking adult female (EF) exploring the yellow hole (right). (**b**) Illustration of the push/lift-up task being used by a high-ranking adult male (WY) using the lift-up option (left) and a low-ranking adult female (EF) using the push option (right). (**c**) Illustration of the rotating-door task being used by a high-ranking adult male (ZA) rotating the door counter-clockwise (left) and another high-ranking adult male (CC) rotating the door clockwise (right).

Three wooden extractive foraging tasks (containing raisins) of increasing difficulty were presented between 4th July to 26th August 2011. The first task (blue/yellow) could be solved, to obtain raisins, by reaching into one of two identical holes painted either blue or yellow. The second task (push/lift-up) consisted of a swing door that could be pushed inwards or lifted up outwards to reach raisins inside. The third task (rotating-door) involved a door that could be spun clockwise or counter-clockwise to uncover a hole through which raisins could be reached (see Fig. 1, and Supplementary IInformation S3 for further detail). The two options for each task led to the same quantity and quality of food rewards. Using prior knowledge^10,20^, the tasks were designed to be of differing difficulties, with blue/yellow task being the easiest and rotating-door task the most difficult. Pilot tests indicated task difficulty was as anticipated.

All tasks were fixed to the ground and were designed such that no moving parts were able to trap hands/digits and no parts were removable. Many raisins were placed inside the tasks at the beginning of each session to avoid causing interference with the natural group dynamics by frequent experimenter refills. When refilling was necessary tasks were obscured and the identity of any monkeys that managed to observe the refilling was noted to control for potential biases.

### Experimental procedure

The tasks were presented, individually, twice per day (morning and afternoon) in the enclosure and rotated around three locations, including feeding sites frequently visited by the macaques, and locations far away from public footpaths. That increased the likelihood that all individuals had opportunity to interact with the tasks. Each task introduction session lasted a maximum of 1 h. Tasks were introduced for a total of 34 days (~ 51 h) in the order of increasing difficulty (blue/yellow 32 h; push/lift-up 12 h; rotating-door 6 h; see Table S1 in Supplementary Information for full details). The duration of task presentation for the first/easy task was longer than for the remaining tasks as it included a familiarisation period for the macaques whom had not experienced such experimental contexts before. Task options were not seeded with trained monkeys.

Task presentations were filmed with one video camera placed at 5 m from the task. CE stood behind this camera, and narrated who was approaching and interacting with the task (Table 1), which options (e.g. push or lift-up door) were used, and who was observing the task interaction within 10 m. Video sessions were coded by IG, and CE coded ~ 30% observed time for inter-observer reliability.

Data on first successful task solution for each individual were used to establish the order and time of acquisition of the novel foraging behaviour. For the purposes of the analysis, it is assumed that an individual has ‘learnt’ the task following their first successful solution. Although we were unable to formally test this, it is a reasonable assumption as several further task solutions tended to immediately follow the first. Data collected during task introductions were also used to calculate a series of individual-level variables that were included in NBDA to control for potential biases, which could indicate social learning when it was not actually present, in the transmission of the novel behaviour (Table 2).

**Table 2:** Task-related interactions used to control for individual-level confounding factors in the network-based diffusion analysis (NBDA) performed in a group of Barbary macaques at Trentham Monkey Forest (UK).

Variable	Definition	Measure/Control for
Contact latency	Time between first approach within 0.5 m of the task (start of a bout) and first physical contact with the task	Normalized continuous variable indicating the degree of fear of the novel task (neophobia)
Contact level	Contact latency transformed into a categorical variable: 1: < 10 s; 2: 10–60 s; 3: 1–3 min; 4: > 3 min	Level of neophobia
Option preference	Category assigned to each of the available options to solve each task Blue/yellow task: 1 = Yellow, 2 = Blue; Push/lift-up task: 1 = Push, 2 = Lift-up; Rotating-door task: 1 = Clockwise, 2 = Counter-clockwise. In all cases, category 3 = No preference	Only when individuals chose one of the options in > 60% of their task interactions, did we consider they showed a preference for that option. This enables measurement of option-bias learning preferences to determine whether not learned preferences, or preferences acquired by own experience of the task or observation of others at task influenced learning rates
Total refills observed	Sum of all the task refilling events for which each individual has been reported as ‘attending’	Controls for learning biases caused by the observation of humans using the options to place rewards inside (Supplementary Information S3)
B/Y proportion refill	Only for blue/yellow task. Total number of refills observed using the blue option divided by the total number of refills observed using the yellow option	Blue/yellow task is the only task where refills needed to be done from two separated parts of the task (the blue and the yellow holes). This controls for biases introduced by observing humans refilling one hole more times than the other. Data shows that individuals either observed both holes being refilled in equal proportion (~ 1) or the blue hole being refilled more times than the yellow hole
Frequency of attention at distance	Number of task interaction bouts individuals observed others interacting with the task but did not approach it (i.e. did not initiate a bout) divided by the total number of bouts individuals were observed within 10 m of the task (not interacting with the task, whether attending or not)	When individuals are interested in the task (attend to the task when this is being used by others) but do not approach the task, it is likely that the task is being monopolised and the observer remains at a distance because it is receiving threats from the demonstrator or is inhibited from approaching the task. This variable controls for monopolisation (i.e., control or domination of the task by one individual)
Frequency of access to the task	Number of bouts where individuals approached within 1 m of the task divided by the total number of bouts individuals were present within 10 m of the task (as demonstrators or observers, interacting or not, attending or not)	This variable indicates variation in the likelihood with which individuals approach the task. It controls for neophobia, monopolisation and other unknown factors. Unknown factors may include contextual factors (empty task, weather conditions, etc.), social factors (presence of dominants in the vicinity, individuals engaged in other social behaviours, etc.) or individual-level factors (motivation, interest, emotional state, etc.)
Rate of performance with the task or transmission of the trait^1^	An individual’s number of successful interactions divided by the total time interacting with the task	Individuals that keep succeeding with the task are reinforced to keep interacting with it, increasing their rate of performance and, thus, potentially influencing transmission of the trait to other group members more than other task-interacting individuals in the group^25,66^

^1^This variable is entered in NBDA as part of the function to calculate non-constant rates of social transmission.

### Statistical analyses

All statistical analyses were performed in R. All p-values obtained in multiple comparisons were adjusted using a Benjamini–Hochberg correction with a False Discovery Rate (FDR) of 5%. Inter-observer reliability was measured using Cohen’s Kappa and interpretation of the level of agreement was made using the rules of Fleiss et al.^67^ and McHugh^68^. There was generally a high degree of inter-observer agreement for identity of monkeys interacting with the task, task options being used, number and type of events or interactions with the task, and identity of observers (Table S4 in Supplementary Information).

#### Network-based diffusion analysis (NBDA)

Both OADA and cTADA (TADA using continuous time) versions of NBDA were used, following guidelines and R codes of Hasenjager et al.^26^. Twelve individual-level variables (ILVs) were included in the NBDA: sex, age, social rank order, social rank class and the eight task-related variables (Table 2). Two agent-based models were generated and compared: a *purely asocial learning model* and an *asocial + social learning model* (the latter tests whether the order/time of diffusion (task solving) follows the pattern of relations of the social network used in the model). Different combinations of ILVs were tested for each model separately using forward selection and backward elimination to find the models with the lowest AICc (best models, Ref.^25^). Variables with a variance inflation factor (VIF) > 4 were considered collinear and removed^69^.

Following guidelines^24–26^ three types of models were tested for each version of NBDA: an additive model (where ILVs only influence asocial learning), a multiplicative model (where ILVs equally influence social and asocial learning) and an unconstrained model (where ILVs differently influence social and asocial learning). Analyses considered constant and non-constant rates of both social transmission of the novel trait (non-constant rate calculated as rate of performance, see Table 2) and asocial acquisition (learning without social influence). Non-constant rates of asocial acquisition were modelled using two baseline rate functions corresponding to a gamma and a Weibull distribution (see Supplementary Information S7). Maximum likelihood methods (AICc and Akaike weights) determined which type of model better explained the observed transmission data. Social transmission was said to occur when the *asocial* + *social learning model* had an AICc value, at least, 2 units lower than the *purely asocial learning model* (ΔAICc > 4 constituted strong evidence and a ΔAICc > 10 very strong evidence for social learning). Evidence of social transmission was also determined by calculating, for each social network and task, the percentage of events that occurred by social transmission (%ST), a likelihood ratio test (LRT) comparing both agent-based models, and the 95% confidence intervals (CI 95%) for the social parameter *s’* which determines the strength of social transmission relative to asocial learning. Finally, the influence of ILVs on social and asocial learning was further investigated for those models that provided evidence of social transmission. For such cTADA models, comparisons with a homogeneous network determined whether social transmission followed the provided network.

#### Task difficulty and analysis of task option preferences

We tested whether the order of task difficulty corresponded to the expected task difficulty using a variety of measures such as learning time and rate of successful and unsuccessful task manipulations (Supplementary Information S5).

Exact multinomial tests (goodness-of-fit analysis) with post-hoc exact binomial tests were conducted to determine group level biases for one task option (e.g. blue or yellow hole) which might influence the transmission of social information. Individuals were classified in three preference categories: (a) showing preference (> 60% use) for option 1, (b) showing preference (> 60% use) for option 2, (c) showing no option preference. A comparative analysis with different threshold percentages was carried out to justify the use of the > 60% criterion (Supplementary Information S6).

To determine option preferences at the individual level, we investigated: (a) *primacy* and *recency effects*: preference for first or most recent option observed in use by a conspecific, respectively (only when both options were observed prior to the individual’s first successful interaction), (b) *copying the most successful*: copying the ‘preferred’ option of the individual observed with the highest proportion of successful manipulations, (c) *frequent exposure*: copying the ‘preferred’ option of the demonstrator most frequently observed in terms of the number of task interaction bouts, (d) *first option*: sticking to the option used on their own first successful manipulation (including those solving the task without prior observation of another solving; henceforth, asocial learners). Effects were measured in observers who manipulated the task > 5 times, and where appropriate (b, c) including only demonstrators/models who were observed for > 5 task manipulations prior to the first successful interaction of the observer. All solvers had observed both options being solved at the end of the task introduction period for each task. Task manipulations were too fast to collect accurate data on who observed whom for every single task interaction. These data, however, were collected as frequently as possible during each interaction bout. That is why we cannot test the influence of overall frequency of choices observed before first own successful interaction with the tasks. Instead, we used ‘frequent exposure’.

## Results

Learning time and rate of successful (but not unsuccessful) manipulations confirmed the tasks increased in difficulty from blue/yellow task > push/lift-up task > rotating-door task, as anticipated (see Supplementary Information S5).

### Network-based diffusion analysis (NBDA)

Results for OADA and cTADA versions are presented in Table 3 and Table S7.1. Outcomes of cTADA matched those of OADA in many cases, however cTADA models sometimes provided different results, in terms of which model (*asocial* versus *asocial* + *social learning*) had a better fit, depending on the baseline acquisition rate function (constant or non-constant) tested (Supplementary Information S7). This suggests that for most cTADA models, the analysis was strongly influenced by the time course of events instead of the network transmission pattern. Accordingly, and following Hasenjager et al.^26^, we use only OADA to make inferences.

**Table 3:** Results for order of acquisition diffusion analysis (OADA) for all the networks and tasks tested in a group of Barbary macaques (*Macaca sylvanus*) at Trentham Monkey Forest (UK).

Task	Network	NBDA	Asocial		Social		ΔAICc	Support	LRT (P)	Approach	Rate of transmission	Rate of acquisition	ILVs	CI95%
			AICc	Akaike	AICc	Akaike								
Blue/yellow	Grooming	OADA	184.30	0.82	187.27	0.18	− 2.97	4.41	0.006 (0.939)	Multi	Non	NA	High class social rank Contact level Number of refills observed B/Y proportion of refills observed Frequency of access to the task	s' = 17.02 (SE = 227.69) Lower = 0, Upper = 772.54
	Huddling	OADA	184.30	0.82	187.27	0.18	− 2.97	4.41	0 (1)	Multi	Non	NA		s' = 0 (SE = 67.74) Lower = 0, Upper = 763.74
	Proximity 1 m	OADA	184.30	0.82	187.27	0.18	− 2.97	4.41	0.005 (0.944)	Multi	Non	NA		s' = 12.57 (SE = 185.51) Lower = 0, Upper = 859.86
	Proximity 5 m	OADA	184.30	0.82	187.27	0.18	− 2.97	4.41	0.029 (0.864)	Multi	Non	NA		s' = 15.38 (SE = 104.55) Lower = 0, Upper = ∞
	Observation 1 m	OADA	184.30	0.74	186.37	0.26	− 2.07	2.81	0.902 (0.342)	Multi	Non	NA		s' = 2.96 (SE = 3.94) Lower = 0, Upper = 15.63
	Observation 5 m	OADA	184.30	0.65	185.55	0.35	− 1.25	1.86	1.723 (0.189)	Multi	Non	NA		s' = 2.61 (SE = 2.73) Lower = 0, Upper = 11.57
	Kinship	OADA	184.30	0.81	187.23	0.19	− 2.93	4.33	0.037 (0.847)	Multi	Non	NA		s' = 1.62 (SE = 8.85) Lower = 0, Upper = 32.57
Push/lift-up	Grooming	OADA	141.23	0.52	141.37	0.48	− 0.14	1.07	2.594 (0.107)	Multi	Con	NA	Contact latency normalized Preferred option^1^ Number of refills observed Frequency of access to the task^2^	s' = 48.29 (SE = 42.97) Lower = 0, Upper = 188.87
	***Huddling***	***OADA***	***141.23***	***0.24***	***138.95***	***0.76***	2.28*	***3.13***	***5.019 (0.025)***	***Multi***	***Non***	***NA***		***s' = 872.96 (SE = 707.97)*** ***Lower = 55.39, Upper = 3574.39***
	Proximity 1 m	OADA	140.87	0.67	142.32	0.33	− 1.46	2.07	1.533 (0.216)	Multi	Non	NA		s' = 685.89 (SE = 848.92) Lower = 0, Upper = 4450.90
	Proximity 5 m	OADA	140.87	0.81	143.78	0.19	− 2.89	4.25	0.090 (0.764)	Multi	Non	NA		s' = 29.68 (SE = 127.75) Lower = 0, Upper = ∞
	***Observation 1 m***	***OADA***	***140.87***	***0.26***	***138.79***	***0.74***	2.08*	***2.83***	***5.069 (0.024)***	***Multi***	***Con***	***NA***		***s' = 1.49 (SE = 1.15)*** ***Lower = 0.10, Upper = 5.41***
	***Observation 5 m***	***OADA***	***141.22***	***0.19***	***138.35***	***0.80***	2.87*	***4.20***	***5.612 (0.018)***	***Multi***	***Non***	***NA***		***s' = 39.98 (SE = 47.81)*** ***Lower = 1.94, Upper = 431.06***
	Kinship	OADA	141.38	0.55	141.81	0.45	− 0.43	1.24	2.089 (0.148)	Multi	Non	NA		s' = 23.88 (SE = 22.87) Lower = 0, Upper = 98.62
Rotating-door	Grooming	OADA	82.48	0.78	85.06	0.22	− 2.58	3.63	0.497 (0.481)	Multi	Con	NA	Contact level Preferred option	s' = 36.42 (SE = 66.65) Lower = 0, Upper = 338.33
	Huddling	OADA	82.48	0.81	85.44	0.19	− 2.96	4.39	0.117 (0.732)	Multi	Con	NA		s' = 8.12 (SE = 27.89) Lower = 0, Upper = 201.43
	Proximity 1 m	OADA	82.48	0.82	85.53	0.18	− 3.04	4.58	0.034 (0.853)	Multi	Con	NA		s' = 3.76 (SE = 21.99) Lower = 0, Upper = 127.63
	Proximity 5 m	OADA	82.48	0.82	85.56	0.18	− 3.07	4.66	0 (1)	Add/Multi	Con/Non	NA		s' = 0 (SE = 5.26) Lower = 0, Upper = 27.12
	***Observation 1 m***	***OADA***	***82.48***	***0.23***	***80.02***	***0.77***	2.46*	***3.42***	***5.536 (0.019)***	***Multi***	***Non***	***NA***		***s' = 219.17 (SE = 246.29)*** ***Lower = 10.29, Upper = 1979.18***
	*Observation 5 m*	*OADA*	*82.48*	*0.27*	*80.52*	*0.73*	1.97*	*2.67*	*5.044 (0.025)*	*Multi*	*Con*	*NA*		*s'* = *1.93 (SE* = *1.63)* *Lower* = *0.12, Upper* = *8.84*
	Kinship	OADA	82.48	0.76	84.75	0.24	− 2.27	3.11	0.807 (0.369)	Multi	Non	NA		s' = 44.31 (SE = 67.69) Lower = 0, Upper = 326.90

*Asocial:* Purely asocial learning model. *Social:* Asocial + social learning model. *ΔAICc:* Difference in AIC between asocial and asocial + social learning models. *Support:* The degree to which the agent-based model (asocial or social) with the lowest AIC is better than the alternative. For example, in the first line the asocial learning model for grooming was 4.41 × better than the corresponding asocial + social learning model for this network. *Approach:* Additive (Add), Multiplicative (Multi) or Unconstrained (Unc) model. *Rate of transmission:* Constant (Con), Non-constant (Non). When models provided the same results using different approaches and rates, LRT and CI 95% were calculated for those with better estimates of the s’ parameter (underlined).

^1^Not included in kinship.

^2^Only included in 1 m & 5 m proximity networks and in 1 m observation networks.

*indicates models that provide evidence of social transmission according to ΔAICc (bold and italics = enough evidence; only italics = almost enough evidence).

For interpretation of CI 95% for the *s’* parameter, refer to Table 1 and SI in Hasenjager et al.^26^.

In all cases, inclusion of neophobia measures improved model fit. Monopolisation measures had a greater effect on the order of acquisition of the trait as task difficulty decreased (Table 3 and Table S7.1). No evidence of social transmission was found for the easiest, blue/yellow task for any of the networks used in the analyses. However, there was evidence of social transmission (∆AICc > 2) in the push/lift-up task when huddling (*social* + *asocial model* 3.13 × more support than *asocial model*) and observation (1 m: 2.83 × support; 5 m: 4.20 × support) networks were used in the analysis, and in the rotating-door task when the 1 m observation network (3.42 × support; Table 3) was used in the analysis.

The likelihood ratio test (LRT) comparing the *asocial* + *social learning* models with their corresponding asocial models confirmed the aforementioned results, with P < 0.05 indicating evidence of an effect consistent with social transmission (Table 3). A significant P value was also obtained for the 5 m observation network in the most difficult, rotating-door task (∆AICc ~ 2). The CI 95% indicated that there was, at least, reasonable evidence for social transmission in the aforementioned models (s’ = 0 not included in the interval). Accordingly, the effect of social transmission could be considered potentially strong in huddling networks, small in 1 m observation networks and of uncertain strength (weak or strong) in 5 m observation networks for the push/lift-up task. The effect of social transmission was small in 5 m, but uncertain in strength in 1 m, observation networks for the rotating-door task (see^26^). LRT and CI 95% support the conclusion of not enough evidence of social transmission for all other models tested.

The percentage of events that occurred by social transmission (%ST) indicated that we can be confident there is evidence of social transmission for huddling and observation networks in the push/lift-up task, and both observation networks in the rotating-door task (0 outside CI 95%; Table 4). Although individuals seemed to use social learning to solve the most difficult tasks, the broad CI95% for %ST suggest that the extent to which they do so is uncertain, except for 1 m observations in the rotating-door task that provides the most robust evidence of social transmission across all models (i.e., narrow CI 95% for %ST; Table 4).

**Table 4:** Contribution of the s’ parameter and ILVs in social learning for models providing evidence of social transmission in a group of Barbary macaques (*Macaca sylvanus*) at Trentham Monkey Forest (UK).

Task	Network	%ST (CI 95%)	ILV	MLE (CI 95%)	Effect (CI 95%)
Push/lift-up	Huddling	41.2 (6.7–60.5)	Contact latency normalized	− 0.68 (− 2.21, − 0.09)	0.51 (0.11, 0.91)
			Preferred option	0.47 (− 0.01, 0.95)	1.60 (0.99, 2.59)
			Number of refills observed	0.18 (0.08, 0.28)	1.19 (1.09, 1.33)
	Observation 1 m	29.8 (5.9–38.9)	Contact level	− 0.49 (− 1.15, − 4.58)	0.61 (0.32, 0.99)
			Preferred option	0.80 (0.24, 1.40)	2.23 (1.27, 4.07)
			Number of refills observed	0.13 (0.03, 0.24)	1.14 (1.03, 1.27)
			Frequency of access to the task	0.55 (0.09, 0.98)	1.73 (1.09, 2.66)
	Observation 5 m	68.3 (15.1–89.2)	Contact latency normalized	− 0.39 (− 2.13, 0.00)	0.68 (0.12, 0.99)
			Preferred option	0.56 (0.06, 1.08)	1.75 (1.07, 2.93)
			Number of refills observed	0.09 (− 0.02, 0.22)	1.09 (0.98, 1.24)
Rotating-door	Observation 1 m	12.4 (5.4–13.2)	Contact level	− 0.88 (− 1.72, − 0.33)	0.41 (0.18, 0.72)
			Preferred option	0.81 (0.17, 1.54)	2.26 (1.19, 4.66)
	Observation 5 m	47.5 (9.5–61.2)	Contact level	− 0.66 (− 1.34, − 0.16)	0.52 (0.26, 0.85)
			Preferred option	0.82 (0.22, 1.50)	2.26 (1.24, 4.48)

*%ST:* Percentage of learning events that occurred by social transmission. *ILV:* Individual level variable. *MLE:* Maximum likelihood estimate of each parameter. *Effect:* The degree to which social and asocial learning increase as measures of the parameters (ILVs) increase, calculated as exp(MLE). For example, in the first line social and asocial learning rates decrease by a factor of 0.51 × per 1 s increase of normalized contact latency. For interpretation of CI95%, refer to Table 1 and SI in Hasenjager et al.^26^.

Finally, all individual-level variables (ILVs) included in the models influenced learning rates (Table 4). Increasing measures of neophobia (contact latency/level) slowed down asocial and social learning rates, whereas an increasing number of task refills observed and frequency of access to the task accelerated both learning rates. Results for preferred options were difficult to interpret (Table 4), so we repeated the analysis breaking down categories into individual variables for the highest supported models (5 m observations for push/lift-up task and 1 m observations for rotating-door task, Table 3). Outcomes suggested that individuals preferring push and counter-clockwise options learned ~ 1.10 × and ~ 1.07 × faster than those using lift-up or clockwise, respectively.

### Task option preferences

For all tasks, multinomial analyses indicated significant differences among preference categories (preference for option 1 or 2 or neither). Post hoc analyses indicated no group-level option preference, nor option preference for apparent asocial learners (Supplementary Information S6). Where data were sufficient to test for primacy and recency effects (blue/yellow and push/lift-up tasks) no effects were found (Table 5).

**Table 5:** Proportion of individuals of a group of Barbary macaques (*Macaca sylvanus*) at Trentham Monkey Forest (UK) that show individual option-preference effects.

Task	Primacy	Recency	Undetermined	Copy most successful	Frequent exposure	Success = frequent	First option
Blue/yellow	5/27 (18%)	8/27 (30%)	14/27^1^ (52%) (N_blue_ = 5, N_yellow_ = 9)	NA	NA	NA	NA
Push/lift-up	5/14 (36%)	2/14 (14%)	7/14^1^ (50%) (N_push_ = 4, N_lift-up_ = 3)	9/19 (47%)	5/19 (26%)	3/19 (16%)	23/28 (82%)
Rotating door	1/6 (17%)	2/6 (33%)	3/6 (50%) (N_clockwise_ = 1, N_counterclockwise_ = 2)	7/9 (78%)	2/9 (22%)	2/9 (22%)	14/16 (88%)

Primacy and recency effects were based on individuals that observed both options being solved prior to first successful interaction. Other effects include all potential social learners (except first option, which also includes asocial learners). *Undetermined:* first and most recent successful option seen were the same.

^1^Five preferred the opposite to the preferred option observed. *Successful = frequent:* preferred option of most frequently, and most successful, individual observed are the same.

#### Blue/yellow task

**Figure 2:**
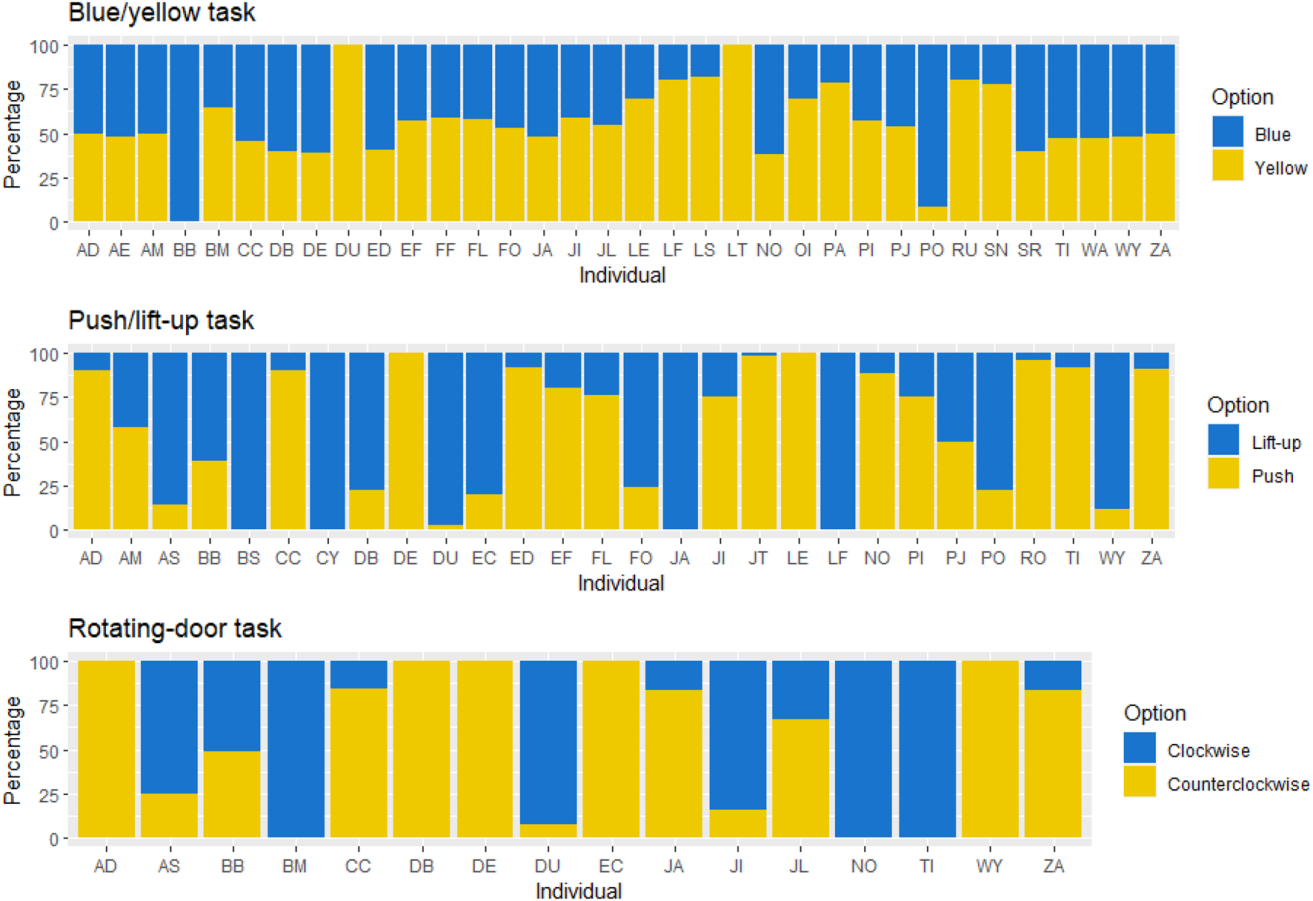
Percentage of options that each individual of a group of Barbary macaques (*Macaca sylvanus*) at Trentham Monkey Forest (UK) used to solve each task. Each bar represents the proportion (in percentage) each individual used each of the two options available in each task, with yellow representing one of the options and blue representing the alternative option.

Thirty-four out of 56 individuals (61%) solved the task at least once, with six of these appearing to do so asocially (asocial learners). Option preferences were almost uniform with a majority of individuals (20 of 34) showing no option preference (Fig. 2). Therefore, no other effects were tested for this task.

#### Push/Lift-up task

Twenty-eight of 56 individuals (50%) solved the task more than once (asocial learners: *N* = 9). Almost half of solvers (~ 43%) showed a preference for the *Lift-up* option (N = 12) and half for the *Push* option (N = 14) indicating individual level option preferences (Fig. 2). As primacy and recency effects did not explain this, other effects were explored. Individuals seemed to retain their first successful option as their preferred option (Table 5).

#### Rotating-door task

Sixteen out of 56 individuals (29%) solved the task at least once (asocial learners: N = 7). Thirty-seven percent (N = 6) of solvers preferred the *Clockwise*, while 56% (N = 9) preferred the *Counter-clockwise,* option (Fig. 2). Individuals seemed to copy the most successful demonstrator observed and tended to retain their first successful option choice (Table 5).

## Discussion

In this study, we used network-based statistical models to investigate the influence of social tolerance and social learning strategies on social learning in Barbary macaques. We first investigated whether social networks depicting different levels of social tolerance predicted social transmission. NBDA indicated that the strength of associations in huddling and observation networks during task introductions predicted the order in which individuals learned the task, contradicting prediction 1a (relating to an influence of kinship) and consistent with predictions 1b (regarding affiliative networks) and 1c (regarding observation networks). For brevity, we will henceforth use the shorthand of ‘X network provided evidence of social learning’ when networks predict the order of trait acquisition in NBDA. In addition, we found evidence of social learning strategies. Consistent with prediction 2a (‘copy when uncertain’/costly information hypothesis) we only found evidence of social transmission for the most difficult tasks (push/lift-up and rotating-door). Finally, for the most difficult task we found evidence of a ‘copy successful’ social learning strategy, consistent with prediction 2b (regarding proficiency bias).

Affiliative relations established via huddling predicted the transmission of social information in one of the tasks (medium difficulty, push/lift-up task). Accordingly, bonds established through socio-positive interactions can represent opportunities of social learning in Barbary macaques^22,23^, as previously reported in other nonhuman primates^27–30^. Grooming, kinship and proximity networks, however, did not predict social transmission. Campbell et al. (2018) found that the selection of huddling partners in Barbary macaques was determined by the strength of grooming relations^70^. Grooming is known to be exchanged for commodities such as social tolerance or access to resources^71,72^. Since huddling has direct social benefits (e.g., thermoregulation) in Barbary macaques^70^, it is likely that grooming is exchanged for huddling (understood as a commodity), as has been suggested for other macaque species^73^. Accordingly, huddling may be more representative than other affiliative relations (i.e. kinship, grooming or proximity) of which individuals have access to certain commodities. Therefore, it is likely that only huddling interactions in our group of Barbary macaques represented those instances of close physical proximity (i.e. high social tolerance) and privileged access to resources (i.e. extractive foraging task), necessary for social learning to occur. This supports the idea that affiliative networks do not always approximate the pathways of social diffusion^59,74^.

For the push/lift-up task (medium difficulty), the huddling network and observation networks showed evidence of social transmission. In addition, *frequency of access to the task* seemed to have influenced social learning for those observing within 1 m. Accordingly, monopolisation probably affected learning rates of close-proximity observers but not of affiliates and observers at 5 m. Such accords with reports that affiliative behaviours lead to higher rates of social tolerance which may result in providing commodities such as priority access to resources in primates^22,71–73,75,76^. Observers situated at 5 m from the task were probably perceived less as competitors by demonstrators since distance in competitive contexts can indicate formal submission^77,78^.

For the rotating-door task (most difficult task), both observation networks showed evidence for social transmission, although it was weaker and less robust for the 5 m than 1 m observation network. This indicates that individuals probably needed to be close to the task in order to learn how to solve it (see below). Note that the 5 m observation network also included observers within 1 m, so the evidence of social transmission provided by this network might well be due to the close-range (1 m) observers. Monopolisation did not seem to have influenced social transmission in the rotating-door task, suggesting that observers/learners were highly tolerated by demonstrators near the task. Our results seem to indicate that as tasks become more difficult, the effect of variables measuring monopolisation becomes weaker or even unfitting to explain social transmission. We argue that individuals might lose interest faster in difficult tasks (reducing monopolisation) since they are more cognitively demanding, or require higher physical prowess and are less rewarding (i.e., less balanced effort-reward trade-off) than easy tasks^79,80^.

Alternatively, it is also possible that in the most difficult task, *frequency of access* (representing monopolisation) had little effect on social transmission as detailed information on action manipulation was required for learning. That is, the level of monopolisation of the task by a demonstrator was irrelevant for social learning to occur when observers were not allowed at a sufficiently close distance during task manipulations to acquire detailed information on how to solve the task. Moreover, those who learned the task obviously had opportunities to access the task, even when they had a low *frequency of access*. Evidence of social transmission in NBDA depends on whether the observation networks used in the model are representative of the type of social learning process/processes occurring^25^. If the task requires a social learning process where detailed information of the actions needs to be observed, it is more likely that evidence for social learning will be found using a network based on observations at a close distance than at a long distance. Moreover, behavioural coordination required for social learning may provide the observer the opportunity to learn general or detailed information from the demonstrator depending on the distance between them^22^. Only the most difficult task (rotating-door) required close proximity to acquire detailed information about demonstrators’ manipulations and payoffs in order to learn how to solve it (discussed further below).

Given the established order of task difficulty (see Supplementary Information S5), our findings align with an increasing reliance on social information as task difficulty increases. Overall, evidence for social learning was obtained for the two most difficult tasks and not the easiest task. This is consistent with ‘costly information’ theory^7^ which states that animals rely on social learning when asocial learning is costly in terms of time and energy investment. Previous studies with other primate species have also found that social learning increases as asocial learning becomes challenging^3,19,20^ or when individuals are naïve^81^.

Faced with a novel/difficult task, individuals may rely on social information if they lack relevant prior knowledge to guide their actions or if they are uncertain about how to use their knowledge in that new/challenging context^3^. It has been suggested that migrant primates conform to group-specific behaviours after immigration likely due to their uncertainty of the new environment (wild vervet monkeys, Ref.^82^; wild chimpanzees, Ref.^83^). In those cases, high uncertainty about new circumstances could pressure individuals to adapt quickly to group behaviour, instead of relying on possibly out-dated or inadequate individual information^83,84^. In our study, each task represented a new challenging context for which knowledge of the previous task was irrelevant/unreliable to solve the following one, and actions became less familiar as task difficulty increased (Supplementary Information S8). Uncertainty regarding how to solve the two most difficult tasks likely led individuals to rely on social information. Accordingly, we have evidence consistent with a ‘copy when uncertain’ social learning strategy in the Barbary macaques.

Analysis indicated that it was only for the most difficult task (rotating-door) that individuals displayed an individual-level option preference. They appeared to be influenced by the rate of success of the individuals they observed plus their own task experience. As there was no effect for the most frequently observed demonstrator, it seems that learners were influenced by the task proficiency of the demonstrator, but not by how frequently they observed specific demonstrators interacting with the task. However, we must caution that results for task option preferences were based on a small sample size and the effect for ‘most frequent demonstrator observed’ was based on interaction bouts instead of counts of single task manipulations.

Results for the rotating-door task align with a ‘copy the most successful demonstrator observed’ social learning strategy (a ‘who’ strategy or ‘indirect bias’)^3,14^. Such model-based strategies have also been reported in other macaque species (Japanese macaques, *Macaca fuscata*, Refs.^46,47,60^; rhesus macaques, *Macaca mulatta*, Ref.^61^; *Macaca sp*., Ref.^62^) and other primates^6,81,85,86^. Note that in our study, Barbary macaques would copy the most successful (proficient) individual (as suggested for long-tailed macaques, Ref.^43^) and not generally successful individuals (i.e. dominants), as seen in captive chimpanzees^81^. As social rank was not included in the best fitted models (Table 3), it had no significant effect on social diffusion in our study. It is likely that only individuals with specific social bonds (not captured in our networks) with demonstrators, irrespective of social rank, were granted close-range access to learn the most difficult task (rotating-door), just as only huddling partners seem to have been privileged learners in the task of medium difficulty (push/lift-up). It is also possible that solving of the rotating-door task involved the evaluation of the payoffs associated with the alternatives presented by the demonstrators’ manipulations^8,43,87,88^ (Supplementary Information S8). This would be deemed a ‘direct bias’ (‘what’ strategy) where individuals adopt the choice they perceived as more valuable or effective^14^. However, our data did not allow us to discriminate between ‘who’ and ‘what’ strategies. The total number of successful task manipulations that each individual observed could not be estimated accurately, and the reward associated with each option was not manipulated.

As observed in other primate species (wild white-faced capuchins, *Cebus capucinus*, Ref.^63^; wild vervet monkeys, Ref.^89^; captive chimpanzees, Ref.^81^), Barbary macaques are influenced by multiple social learning strategies simultaneously (i.e. ‘copy when uncertain’, ‘copy most successful demonstrator’).

To conclude, we have highlighted evidence of reliance of Barbary macaques on social learning, influenced by social tolerance, task difficulty and model-success, which supports the costly information hypothesis and several social learning strategies. This is the first study that provides quantitative evidence of social learning in Barbary macaques, and one of the first studies using statistical methods to directly evidence social transmission in macaque species. Our data analysis also provides tentative information regarding social learning processes (Supplementary Information S8) and contributes evidence on animal culture from the genus that sparked the animal culture debate in the 1950s. Further research is necessary to unravel which cognitive mechanisms, and learning biases characterise social learning in macaques. Such analyses may benefit from the integration of multi-layer^90–93^, and dynamic^5,59^, networks in NBDA.

## Supplementary Information

Supplementary Information.

## Data Availability

The datasets generated during the current study are available from the corresponding author on reasonable request.
