# Supplementary material for: The influence of task difficulty, social tolerance and model success on social learning in Barbary macaques
Source: Sci Rep. 2023 Jan 20;13:1176. doi: 10.1038/s41598-022-26699-6 (PMC9860066; doi:10.1038/s41598-022-26699-6)
Supplement: Supplementary file 1 — Supplementary Information. [file 41598_2022_26699_MOESM1_ESM.pdf]

SUPPLEMENTARY INFORMATION FOR

The influence of task difficulty, social tolerance and model success on social learning in Barbary macaques

Ivan Garcia-Nisa*, Cara Evans & Rachel L. Kendal

S1 – Order and duration of task introductions

Table S1. Task-introduction schedule for a group of Barbary macaques (*Macaca sylvanus*) at Trentham Monkey Forest (UK)

	**Day 1 to 14**		**Day 15 to 24**		**Day 25 to 30**		**Day 31 to 34**		
**Task**	AM	PM	AM	PM	AM	PM	AM	PM	**Total days (time)**
Blue/yellow task (easy)	✓	✓	✓						23d (32h 25’)
Push/lift-up task (intermediate)				✓	✓				14d (12h 39’)
Rotating-door task (difficult)						✓	✓	✓	10d (06h 13’)

S2 – Social rank analysis

Data on agonistic encounters were collected using behavioural measures based on agonistic competitions and formal dominance to calculate dominance ranks [1]. Agonistic competitions are referred as dyadic interactions where one subject (the winner) directs an agonistic behaviour (e.g. hit, slap, bite, threat) towards another subject (the loser) who displays a submissive behaviour (e.g. flee, silent-bared teeth display, submissive grin, see ***Table 1*** in ***Main Text***). These agonistic encounters are characterized by the asymmetry of the outcome (i.e. win or lose) [2]. On the other hand, lack of aggressiveness/agonism refers to those instances where conflicts are resolved using non-agonistic assessments (i.e. submissive behaviour) and escalated fights do not take place [2]. In these contexts, it is assumed that one subject (the subordinate) has learned from previous encounters with a conspecific or recognizes some dominance features on its opponent that bias its behaviour towards fleeing-upon-approach responses or submission/yielding when receiving threats [3]. Therefore, the subordinate recognizes its inferior position, so the dominance relationship is readily accepted instead of agonistically challenged. This dominance context is termed ‘formal dominance’ [1].

Dominance ranks can be calculated using different statistical methods. Here, we followed the recommendations of Funkhauser et al. (2018) [1] for calculating dominance ranks using different approaches, correlate the scores obtained across methods and, if there are no significant differences,

calculate median ranks across all these ranking procedures, which minimizes errors and takes conservative interpretations of dominance hierarchy with minimal data.

Following Funkhauser et al. (2018) [1], we measured dominance ranks using five ranking methods: a) **I&SI method,** a non-parametric method that uses a numerical criterion that is maximized or minimized in the re-organization of a data matrix of dominance relations (i.e. dominance matrix, b) **David's scores**, that calculates the individual overall success and ranks the subjects in order according to this measure, c) **Elo-ratings**, a non-matrix based technique to determine dominance ranks assuming linearity [4], d) **ADAGIO** [5] and e) **PERC** [6], two different methods that analyse dominance without making structural assumptions of the hierarchy (i.e. network-based methods). More information about the hierarchy methods used in this study can be found in ***Table S2.1*** and in Funkhauser et al. (2018) [1]. Finally, we used Spearman rank correlations with Benjamini-Hochberg corrections to determine the reliability across rankings provided by the different methods.

Table S2.1. Description of the methods and calculations used to determine dominance hierarchies in a group of Barbary macaques (*Macaca sylvanus*) at Trentham Monkey Forest (UK).

I&SI method	
Description	The method is based in the re-organization of individuals assuming a linear hierarchy by minimizing the number of inconsistencies (I) and the total strength of inconsistencies (SI) in a matrix of dominance relations [7][8][9].
Calculations	DomiCalc was used, which is a series of Excel macros (the script is the same applied in R by Leiva et al., 2010 [10] using the ISI.method function). In contrast to the R function, DomiCalc provides all the alternative optimal ranking solutions and uses the differences between numbers of dominations and subordinations (Dom-Sub) and the proportion of dominations (PD) in the last step of the procedure to break ties and decide the final ranking order (optimization of the method) [9]. Linearity was measured using the improved Landau h' test with the linear.hierarchy.test function in R.
David's scores	
Description	The method derives a dominance index based on the overall success of each individual and the relative strenght of its opponents. It calculates the proportion of wins over losses in agonsitic encounters relative to the total number of observed interactions and corrected for chance occurrences of observed outcomes [11][12].
Calculations	The R function steeptest (package 'steepness') was used, which calculates steepness and derives David's scores using the improved algorithm with the correction of chance probabilities suggested by Gammell et al. (2003) [12]. Normalized and non-normalized David's scores were estimated. Steepness of a dominance hierarchy refers to the size of the absolute differences between adjacently ranked individuals and their overall success in winning dominance encounters. Steepness is the absolute slope of the straight line fitted to the normalized David's scores plotted against individual ranks [13].
Elo-ratings	
Description	The method provides sequential estimations of individual dominance strengths based on the actual sequence of dominance interactions. It is based on the assumption that the chance of individual A winning B is a function of the difference in current ratings of the two contestants. Therefore, the method takes into account the sequence of interactions and updates the rating of each individual after each contest until the last contest observed to provide the final ranking

	scores [4][14].
Calculations	The R package 'EloOptimized' was used to calculate Elo-ratings using the traditional method (eloratingfixed function) and the optimized method (eloratingopt function). The traditional method considers the parameter k (which determines the number of rating points that an individual wins or loses after each encounter) as constant and assigns the same initial Elo-scores to all the individuals (1000 by default). The optimized method uses the maximum likelihood approach to calculate the values of k that better fit the data and the initial Elo-ratings (using AIC measures of model fit). Rank stability was measured using the ratio of rank changes per individuals present over a given time period [15]. The stability index is formally expressed as: $S = \frac{\sum_{I=1}^{d}(C_i\, x\, w_i)}{\sum_{i=1}^{d} N_i}$ where C_i is the sum of absolute differences between rankings of two consecutive days, w_i is a weighting factor determined as the standardized Elo-rating of the highest-ranking individual involved in a rank change, and N_i is the number of individuals present on both days.
PERC (Percolation-conductance)	
Description	The method is a network-based ranking model. It attempts to determine dominance ranks of individuals in a group under the assumption that the hierarchy structure may not be linear, so dominance relations are not completely transitive, meaning that if A dominates B and B dominates C, A does not necessarily dominates C [6]. The method follows two main steps. First, a series of matrices are calculated based on pairwise interactions plus transitive dominance inferred from interactions with common third-parties in order to estimate dominance potential probabilities. Then, individuals are assigned a rank according to the final matrix calculated in step one and a simulated annealing algorithm (see [6]) is used to minimize the number of inconsistencies to provide a final rank (confident bounds of individual ranks are derived).
Calculations	The R package 'Perc' and the guidelines developed by **Fushing & McCowan labs** were used. Calculations were based on a matrix that combines information from direct win/loss interactions with information from indirect pathways between individuals to calculate a matrix of probabilities where each row individual outranks the column individual. The analysis provides heat maps of the individual ranks to identify non-linear dominance structures and takes into account the uncertainty of the data due to potential intransitivities. The annealing algorithm seeks to minimize the costs of potential inconsistencies due to these intransitivities by re-ordering the matrix (with values of dominant subjects above the diagonal and values of subordinate subjects below the diagonal) so that

	the total sum of the probability values below the diagonal is reduced to the maximum [6].
ADAGIO (Approach for Dominance Assessment in Gregarious species)	
Description	This method represents dominance hierarchies using directed acyclic graphs (DAG). A DAG is a network free of cycles (i.e. there is no path from one node back again to the same node). This method does not assume linearity (but it can detect it) and assumes there are no circular triads and that dominance relations are irreflexive (no individual is dominant over itself), transitive (if A>B and B>C, then A>C) and asymmetric (if A>B, B cannot be > A). ADAGIO uses dyadic dominance relationships to extract a dominance hierarchy in the form of a DAG for a given dominance network. If the network is not a DAG, ADAGIO transforms the network into a DAG by detecting the largest strongly connected components (cycles) and extracting the weakest link of this cycle. ADAGIO predicts linear and non-linear hierarchies much better than other methods. Also, observational zeros do not influence the performance of the analysis, and it is so versatile that it can be used for large groups where most relationships are unknown and for small (N < 6) sample sizes [5].
Calculations	The **Adagiov1.1 package (ADAGIO Release 1.1)** provided by Douglas et al. (2017) was used [5]. This package uses a script performed in the Windows command line and Java 1.8. I performed the four versions of the analysis: a) no pre-processing + bottom-up approach, b) no pre-processing + top-down approach, c) pre-processing + bottom-up approach and d) pre-processing + top-down approach. Pre-processing allows breaking symmetry at the dyadic level: for a given dominance, the weight (strength) of the edge between two individuals, where one appears to be dominant to the other, is set to the difference in the number of interactions won by the dominant one, and the reciprocal edge is removed. In the case of a tie, both edges are not considered in the analysis. Top-down and bottom-up approaches serve to compute ranks. The top-down approach sets the ranks of all the roots of the network at 1 (roots = individuals that give but do not receive edges), and the ranks of all the other subjects depend on how many edges they receive +1. The bottom-up approach is the converse method and it starts with leaves (nodes that are not the source of any edge). Different measures are used to decide which ADAGIO version provides better results: a) relative error (proportion of number of interactions removed after pre-processing), b) weight (strength of the relation) and edges removed (proportion of weight or edges of dominance relations removed, respectively, due to pre-processing and DAG conversion), c) Directional Consistency Index (DCI) as estimated by van Hooff & Wensing (1987) [16] which reflects the frequency with which the behaviour occurred in its more frequent direction relative to the total number of times the behaviour occurred.

A total of 828 agonistic encounters were used in the hierarchy analyses. Linearity was low and significant (h' = 0.112, p<0.001), as well as steepness (stp = 0.049, p<0.001, ***Figure S2.1***), as expected in Barbary macaques which are intermediate in the despotic-egalitarian spectrum [17][18].

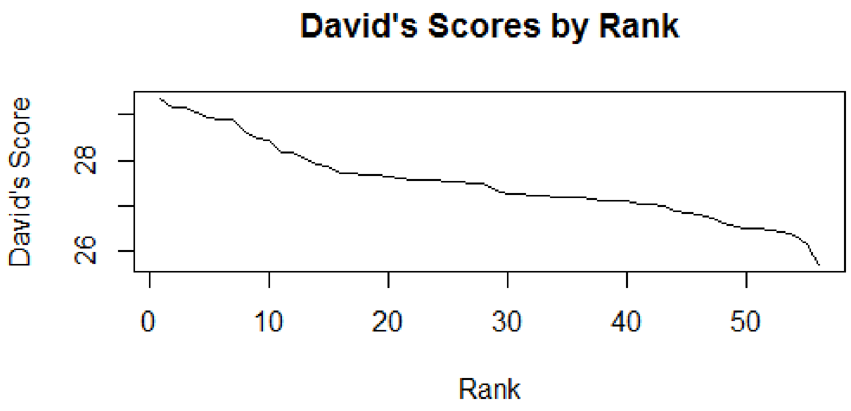

Figure S2.1. Normalized David's scores plotted against individual ranks of a group of Barbary macaques (*Macaca sylvanus*) at Trentham Monkey Forest (UK). The continuous line represents steepeness of dominance relations.

Only one optimal ranking order was obtained for the I&SI method (I: 6, SI: 62). Elo-ratings were calculated using the optimized algorithm with a burn-in period of 100 interactions. On the first 100 interactions, only information of social rank for 31 out of 56 individuals was available (55% of the group). Therefore, we calculated stability from the moment information on dominance relations for all individuals was available until the end of the study (a total of 45 days). Results indicate that the hierarchy is somehow stable but changes in dominance relations are not rare (rank differences = 1165, S = 0.397). This may be due to the sample size, since the group is large and the complexity of the environment they live in may have hindered the observation and occurrence of agonistic encounters.

PERC resulted in 642 transitive triangles, 11 intransitive triangles and a transitivity value of 0.98, indicating that dominance relations were linear. Since some intransitivities were present, some optimal solutions of the PERC simulations have greater costs than others (cost range: 54.99 – 67.62). Therefore, the best ranking order is the one provided by the simulation with a lower cost value. Uncertainty measures indicated that 14.8% of all possible dyads (458 out of 3080) were not clearly defined (i.e. uncertainty probability <0.60, ***Figure S2.2***). Again, the large and complex environment and group size of this group of Barbary macaques makes highly likely that most of the possible dyadic interactions did not take place or were so infrequent that could rarely be observed. This may have caused that some dominance relations were not well established and even changed depending on a different audience at the moment of the encounter.

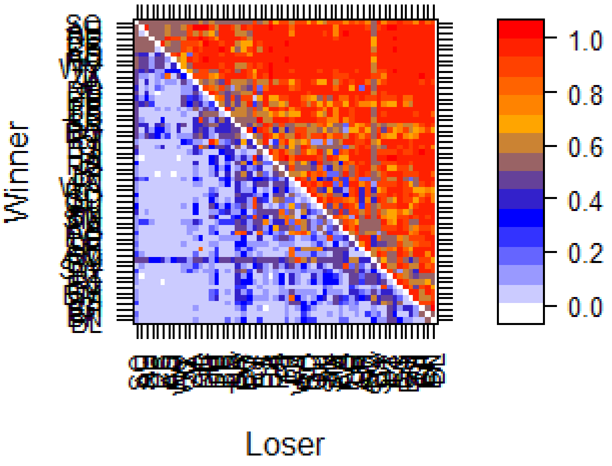

Figure S2.2. Heat map of dominance probabilities between winners and losers in a group of Barbary macaques (*Macaca sylvanus*) at Trentham Monkey Forest (UK). Individuals in the diagonal with values closer to 0.5 indicate uncertain relationships.

Due to the presence of intransitivities and the small sample size, pre-processing approaches were used in ADAGIO to break symmetries and potential ties of dominance relations at the dyadic level [5]. DCI (DCI=0.963, see ***Table S2.1***) indicated that data mostly contained unidirectional relations, meaning that in most cases, the dominance relationships between individuals were well-defined and there were few cases of tied dominance relations [5]. Both top-down and bottom-up approaches led to the same ranking order.

Results of the ranking orders obtained for all methods can be seen in ***Table S2.3***. Correlations among ranking methods were high and significant in all cases (***Table S2.4***). Since all methods were equally reliable, a median of ranks across methods was calculated and used in this study as a measure of ordinal social ranks. Jenks Natural Breaks Classification was used as a measure of categorical social ranks. Jenks Natural Breaks Classification is an optimization method designed to determine the best arrangement of a set of values into different classes or categories [19]. In this case, the method uses social ranks established via hierarchical analyses and finds the best arrangement to split the ranks into a pre-defined number of classes (i.e., high, middle and low ranking classes). This is done by minimizing the average deviation from the class mean (i.e., reducing the variance within classes) while maximizing the deviation from the means of the other classes (i.e., maximizing the variance between classes). The method is commonly used in studies of animal dominance ranks [20][21][22] and already implemented in R packages for hierarchical analysis [23].

Table S2.3. Dominance ranks obtained using all ranking methods in a group of Barbary macaques (Macaca sylvanus) at Trentham Monkey Forest (UK).

Individuals	I&SI method	DS	Elo ranks	PERC	ADAGIO	Jenks Natural Breaks Classification	Median ranks all methods
DE	1	2	2	4	1	high	2
CC	2	5	1	5	2	high	2
ZA	3	1	9	11	3	high	3
AD	4	3	7	7	2	high	4
NO	5	4	6	8	3	high	5
ED	6	6	4	6	3	high	6
AE	7	8	3	2	3	high	3
RU	8	9	10	3	4	high	8
WY	9	7	5	9	3	high	7
JT	10	10	8	10	3	high	10
SR	11	11	12	19	6	high	11
SQ	12	19	17	1	5	mid	12
JL	13	15	15	12	5	high	13
BO	14	26	23	21	6	mid	21
DB	15	14	11	13	5	high	13
BB	16	12	16	17	6	mid	16
TI	17	13	13	14	4	high	13
LS	18	23	29	18	7	mid	18
BS	19	30	37	24	7	low	24
TW	20	21	27	20	4	mid	20
JA	21	24	26	26	6	mid	24
OI	22	22	22	23	4	mid	22
DU	23	16	14	15	5	high	15
FF	24	17	19	16	6	mid	17
ET	25	20	25	22	4	mid	22
JI	26	18	28	25	5	mid	25
PJ	27	25	31	30	9	mid	27
PI	28	29	21	28	10	mid	28
WA	29	39	32	31	7	mid	31
LE	30	38	40	41	12	low	38
RO	31	44	35	32	8	mid	32
LF	32	47	46	29	8	low	32
SD	33	41	36	33	12	mid	33
QU	34	36	30	34	7	mid	34
SM	35	31	20	37	13	mid	31
RS	36	34	34	27	12	mid	34
FL	37	37	44	35	8	low	37
AM	38	51	49	43	11	low	43
SN	39	32	41	36	11	low	36
AS	40	43	42	39	13	low	40
BY	41	27	18	45	13	mid	27
SF	42	45	47	42	13	low	42
FO	43	33	33	44	10	mid	33
SW	44	35	38	46	11	low	38
PA	45	28	24	38	10	mid	28
PO	46	52	45	40	9	low	45
LT	47	54	51	48	8	low	48
EC	48	55	54	49	9	low	49
CY	49	56	56	50	10	low	50
BZ	50	40	48	47	11	low	47
BM	51	50	39	51	12	low	50
SS	52	42	43	52	11	low	43

GI	53	46	50	53	12	low	50
EF	54	53	52	54	12	low	53
BL	55	48	55	56	13	low	55
BN	56	49	53	55	13	low	53

DS rank: Rank obtained using David's scores. Elo ranks: Rank obtained using Elo-ratings. Median ranks all methods: Median of ranks calculated across all ranking methods.

Table S2.4. Correlations of dominance ranks between ranking methods calculated for a group of Barbary macaques (*Macaca sylvanus*) at Trentham Monkey Forest (UK).

Correlations	**Spearman's rho**
I&SI - David's scores	rho = 0.9084 p = < 0.001
I&SI - Elo-ratings	rho = 0.8918 p = < 0.001
David's scores - Elo-ratings	rho = 0.9513 p = < 0.001
PERC - I&SI	rho = 0.9666 p = < 0.001
PERC - David's scores	rho = 0.9033 p = < 0.001
PERC - Elo-ratings	rho = 0.8923 p = < 0.001
ADAGIO - I&SI	rho = 0.8713 p = < 0.001
ADAGIO - David's scores	rho = 0.8175 p = < 0.001
ADAGIO - Elo-ratings	rho = 0.7809 p = < 0.001
ADAGIO - PERC	rho = 0.8764 p = < 0.001

S3 – Description of the tasks

Three extractive foraging tasks of increasing difficulty (with raisins used as rewards) were presented to a group of Barbary macaques.

Task 1: Blue/yellow task

The blue/yellow task consisted of a rectangular wooden box 28 (w) x 16 (h) x 16 (d) cm with two option holes in the top (6 x 6 cm), one framed in yellow and another framed in blue (***Figure 1A*** in ***Main Text***) inspired by a task used by Kendal et al. (2005) [24] with callitrichid monkeys. These colours were chosen because they are equally visible to di and tri-chromatic individuals. Between the two holes, inside the box, two connected pendulum doors hung (***Figure 1A*** in ***Main Text***). When a monkey introduced its hand inside one of the holes, the pendulum on that side was pushed to the centre of the box causing the other pendulum to covering the other hole. This mechanism prevented both holes from being used simultaneously to retrieve rewards. When necessary, the task was refilled using the two option holes. The task was fixed to the ground using long U-shaped metal anchor stakes.

Colours were used to distinguish both options. Afro-Eurasian primates like Barbary macaques have trichromatic vision [25][26][27], so they are capable of distinguishing blue from yellow, green or red. Only a preference for red items has been found in macaque species [28], supporting the foraging

hypothesis, that states that trichromatic vision is an adaptation to facilitate visual detection of ripe fruit [29][30]. In order to prevent colour biases, red was avoided in the tasks.

Task 2: Push/lift-up task

The push/lift-up task was also inspired by a task used by Kendal et al. (2005) [24] and consisted of a wooden square-box 21 (w) x 21 (h) x 21 (d) cm with a swing door at the front that could be pushed inward or lifted up outward (***Figure 1B*** in ***Main Text***). A 3-cm gap between the bottom of the door and the bottom of the box allowed monkeys to manipulate the swing door. The task was refilled through a hole in the back that was covered with a wooden lid screwed to the box. By unscrewing one of the two screws in the lid, the researcher could swing the lid to one side and refill the task. The raisins were placed at the back of the box. The task was attached to a metal cylinder that was already fixed to the ground in the enclosure.

Task 3: Rotating-door task

The rotating-door task was inspired by a task used with wild lemurs [31], and consisted of a squared-wooden box 23 (w) x 23 (h) x 23 (d) cm with a circular retrieval hole (8 cm in diameter) that was covered by a circular door (9.5 cm in diameter) that could be rotated clockwise or counter-clockwise (***Figure 1C*** in ***Main Text***). By rotating door, monkeys could uncover the hole that gave access to the raisins. Once uncovered, the monkeys could stretch their arms through the retrieval hole to reach the raisins placed inside at the bottom of the box. The task was refilled using the circular retrieval hole. The task was fixed to the ground using long U-shaped metal anchor stakes.

S4 – Inter-observer reliability

Table S4. Measures of agreement for inter-observer reliability using Cohen's kappa for data collected on a group of Barbary macaques (*Macaca sylvanus*) at Trentham Monkey Forest (UK).

Variable	Cohen's Kappa	ASE	p-value	CI5%	CI95%	Level of agreement	
						Fleiss et al. (2003) [32]	McHugh (2012) [33]
**Monkey identity**	U: 0.99<br>W: 0.99	U: 0.003<br>W: 0.006	U: 0 (z: 336.8)<br>W: 0 (z: 161.4)	U: 0.99<br>W: 0.98	U: 1<br>W: 1	Excellent	Almost perfect
**Action performed**	U: 0.96<br>W: 0.98	U: 0.012<br>W: 0.009	U: 0 (z: 81.95)<br>W: 0 (z: 111.64)	U: 0.94<br>W: 0.96	U: 0.98<br>W: 0.99	Excellent	Almost perfect
**Events**	U: 0.88<br>W: 0.89	U: 0.014<br>W: 0.013	U: 0 (z: 64.11)<br>W: 0 (z: 66.83)	U: 0.85<br>W: 0.86	U: 0.91<br>W: 0.92	Excellent	Strong
**Who observes whom**	U: 0.70<br>W: 0.66	U: 0.046<br>W: 0.059	U: <0.001 (z: 15.26)<br>W: <0.001 (z: 11.01)	U: 0.61<br>W: 0.54	U: 0.79<br>W: 0.77	Good	Moderate

ASE: Assymetric Standard Error. **p-value:** null hypothesis = agreement is the same as chance agreement (kappa = 0), alternative hypothesis = agreement is different from chance agreement (kappa ≠ 0). **CI:** confidence intervals.

Both unweighted (U) and weighted (W) versions of Cohen's kappa were measured. The difference between unweighted and weighted kappa is that weighted kappa incorporates the magnitude of each disagreement and provides partial credit for disagreements when agreement is not complete [34].

Sessions coded by CE included missing and additional information on who observed whom that were not available when IG coded the videos. This difference might explain the lowest level of agreement for this variable (***Table S4***), which does not make the data coded on who observes whom less reliable but more conservative.

S5 – Confirming task difficulty

Methods:

For each individual, we calculated (i) learning time (time of first contact with the task – time of first task solution/success) and (ii) rate of successful and unsuccessful manipulations (i.e. number of successful or unsuccessful manipulations divided by time spent within the 0.5m area of task). Measurement of learning time controls for time to first contact, so differences should not be attributed to variation in task salience or neophobia. According to normality results using Shapiro-Wilk tests (see Supplementary S5), non-parametric analyses (Kruskal-Wallis and post-hoc Dunn tests with Benjamini-Hochberg correction) were used to test for task difficulty. First, we compared the number of successful manipulations, given the duration of presentation, among tasks. Then, we compared the frequency of successful versus unsuccessful manipulations within each task for the whole group and for a subset of asocial learners (i.e. innovators who solved the task prior to observing its solution). Finally, we compared learning time among tasks.

Results:

The rate of successful manipulations differed among the three tasks (Kruskal-Wallis $\chi^2 = 20.585$, df = 2, $p < 0.001$), with a higher rate of successful manipulations in the blue/yellow task than in the push/lift-up (Dunn test: $p = 0.013$) and the rotating-door (Dunn test: $p < 0.001$) tasks, and in the push/lift task than in the rotating-door task (Dunn test: $p = 0.009$). The rate of unsuccessful manipulations was significantly different among tasks (Kruskal-Wallis $\chi^2 = 37.741$, df = 2, p-value < 0.001) with higher rates in the blue/yellow task than in the push/lift-up task (Dunn test: $p < 0.001$) and the rotating-door task (Dunn test: $p < 0.001$), and no difference between these two latter tasks (Dunn test: $p = 0.288$). Only the rate of successful task manipulations indicates that task difficulty varied as anticipated (see *Discussion*).

For all tasks, individuals performed significantly more successful than unsuccessful manipulations. Amongst apparent asocial learners, individuals performed more successful than unsuccessful interactions for push/lift-up and rotating-door tasks only (***Table S4***).

Table S5. Kruskal-Wallis tests comparing the frequency of successful and unsuccessful manipulations per task in a group of Barbary macaques (*Macaca sylvanus*) at Trentham Monkey Forest (UK).

	**Task**	**Estimate (median)**	**Statistic (V)**	**CI5%**	**CI95%**	**p-value**
**All individuals**	Blue/yellow	-0.025	126	-0.044	-0.007	0.006*
	Push/lift-up	-0.049	13	-0.062	-0.037	< 0.001*
	Rotating-door	-0.023	31	-0.039	-0.009	0.019*
**Asocial learners**	Blue/yellow	-0.006	10	-0.051	0.037	1
	Push/lift-up	-0.056	0	-0.082	-0.031	0.004*
	Rotating-door	-0.043	0	-0.131	-0.018	0.016*

CI: confidence intervals *: indicates significant results

Finally, differences in learning time among tasks were not significant (Kruskal-Wallis χ^2 = 4.225, df = 2, p-value = 0.12). However, post-hoc tests revealed a significantly longer learning time for the rotating-door task compared to push/lift-up (Dunn test: p = 0.048) and the blue/yellow (Dunn test: p = 0.089) tasks, although the latter only approached significance.

Discussion:

Tasks were designed to be of increasing difficulty: blue/yellow task (low), push/lift-up task (medium), rotating-door task (high). Outcomes confirmed that the rotating-door task required more learning time and, therefore, was more difficult than the push/lift-up and blue/yellow tasks, as expected. Also, the rate of successful manipulations indicated that blue/yellow task was the easiest task and rotating-door task was the most difficult of the three. These results were inconsistent with those obtained when the rate of unsuccessful manipulations was considered, for which the blue/yellow task appeared to be more difficult than the other tasks. However, introductions of the blue/yellow task presented a series of flaws that affected the successful retrieval of raisins but not the manipulation of the actions (consisting in stretching one hand through a hole, a component that was also present in the other tasks). These problems included: a) an internal mechanism of pendulum doors that hindered the extraction of rewards, b) the impossibility to know when the task was empty, causing monkeys to attempt to solve it when success was not possible, c) a period of habituation to extractive foraging tasks, a novel context for this group of monkeys. All these issues, that most likely increased the number of unsuccessful manipulations and latency to first success, were solved and/or not observed in push/lift-up and rotating-door tasks.

Finally, the blue/yellow task was identical to the round-box task used by Kendal et al. (2009) [35] with callitrichids, the easiest task tested in their study, in which they used a more difficult task (flip-top box) similar to our push/lift-up task. In conclusion, we determine that blue/yellow task was the easiest task of this study, push/lift-up task was of medium difficulty and rotating-door was the most difficult task tested.

S6 – Multinomial analysis and comparative analysis of threshold percentages for option preference

We tested whether individuals showed a preference for one of the two available solving-options in each task using an exact multinomial analysis. The analysis was based on considering that individuals showed a preference for one option if the number of times they used that option (calculated as the percentage of use of that option) was above a threshold. We used multinomial analysis to test option preferences using different percentages of use to establish that threshold value. Options used a % of times above the threshold were considered the individual's task-option preference. If none of the options was used a % of times above the threshold considered in each case, individuals were considered to have 'No preference' for any of the task options available. Results of the exact multinomial tests can be seen in ***Tables S6.1*** to ***S6.3***.

Table S6.1. Exact multinomial test results for the blue/yellow task presented to a group of Barbary macaques (*Macaca sylvanus*) at Trentham Monkey Forest (UK).

Criteria	Blue	Yellow	No preference	pObs	p-value	Post-hoc (p-value)
**>50%**	15	18	1	0.0000	0.0001*	Y-B: 0.72833<br>Y-N: 0.00023*<br>B-N: 0.00078*
**>55%**	7	15	22	0.0004	0.0196*	Y-B: 0.201<br>Y-N: 0.324<br>B-N: 0.024*
**>60%**	4	10	20	0.0001	0.0032*	Y-B: 0.1796<br>Y-N: 0.1481<br>B-N: 0.0046*
**>65%**	2	9	23	0.0000	0.0000*	Y-B: 0.065<br>Y-N: 0.030*<br>B-N: 5.8e-05*
**>70%**	2	7	25	0.0000	0.0000*	Y-B: 0.1797<br>Y-N: 0.0032*<br>B-N: 1.7e-05*
**>75%**	2	7	25	0.0000	0.0000*	Y-B: 0.1797<br>Y-N: 0.0032*<br>B-N: 1.7e-05*
**>80%**	2	3	29	0.0000	0.0000*	Y-B: 1.00<br>Y-N: 3.8e-06*<br>B-N: 1.4e-06*

Number of individuals using each option: B = Blue option. Y = Yellow option. N = No preference. pObs: multinomial probability for the observed counts and probabilities. The post-hoc column informs about the p-values after pair-wise comparisons and Benjamini-Hochberg correction. *: indicates significant results.

Table S6.2. Exact multinomial test results for the push/lift-up task presented to a group of Barbary macaques (*Macaca sylvanus*) at Trentham Monkey Forest (UK).

Criteria	Push	Lift-up	No preference	pObs	p-value	Post-hoc (p-value)
**>50%**	15	12	1	0	0.0006*	L-P: 0.7011<br>L-N: 0.0051*<br>P-N: 0.0016*
**>55%**	15	12	1	0	0.0006*	L-P: 0.7011<br>L-N: 0.0051*<br>P-N: 0.0016*
**>60%**	14	12	2	0.0002	0.0063*	L-P: 0.845<br>L-N: 0.019*<br>P-N: 0.013*
**>65%**	14	11	3	0.0006	0.0197*	L-P: 0.690<br>L-N: 0.086*<br>P-N: 0.038*
**>70%**	14	11	3	0.0006	0.0197*	L-P: 0.690<br>L-N: 0.086*<br>P-N: 0.038*
**>75%**	12	11	5	0.0058	0.2228	NA
**>80%**	10	7	11	0.0183	0.7041	NA

Number of individuals using each option: P = Push option. L = Lift-up option. N = No preference. pObs: multinomial probability for the observed counts and probabilities. The post-hoc column informs about the p-values after pair-wise comparisons and Benjamini-Hochberg correction. *: indicates significant results. NA: Not applicable (exact multinomial test not significant).

Table S6.3. Exact multinomial test results for the rotating-door task presented to a group of Barbary macaques (*Macaca sylvanus*) at Trentham Monkey Forest (UK).

Criteria	Clockwise	Counter-clockwise	No preference	pObs	p-value	Post-hoc (p-value)
**>50%**	7	9	0	0.0003	0.0057*	W-C: 0.804<br>W-N: 0.012*<br>C-N: 0.023*
**>55%**	6	9	1	0.0019	0.0336*	W-C: 0.607<br>W-N: 0.064*<br>C-N: 0.188
**>60%**	6	9	1	0.0019	0.0336*	W-C: 0.607<br>W-N: 0.064*<br>C-N: 0.188
**>65%**	6	9	1	0.0019	0.0336*	W-C: 0.607<br>W-N: 0.064*<br>C-N: 0.188
**>70%**	6	8	2	0.0084	0.1596	NA
**>75%**	5	8	3	0.0167	0.3445	NA
**>80%**	5	8	3	0.0167	0.3445	NA

Number of individuals using each option: C = Clockwise option. W = Counter-clockwise option. N = No preference. pObs: multinomial probability for the observed counts and probabilities. The post-hoc column informs about the p-values after pair-wise comparisons and Benjamini-Hochberg correction. *: indicates significant results. NA: Not applicable (exact multinomial test not significant).

In general, results showed that except for 50% (an extremely optimistic threshold) in the blue/yellow task, outcomes were not overly sensitive to which preference threshold criteria was used. Although option preferences for each category did not differ among the % thresholds tested for the rotating-door task (see ***Table S6.3***), the exact multinomial test only did not detect differences among preference categories for the three most conservative values (>70%, >75% and >80%). In general, results indicate that the 60% criterion for option preferences used in this study is an intermediate value (not too optimistic like 50% nor too conservative like 80%) that allowed detection of option preferences in all tasks.

Using the 60% criterion, the exact multinomial tests showed no option preference for asocial learners in any of the tasks (**blue/yellow task:** N=6, pObs= 0.041, p-value = 0.383; **push/lift-up task:** N=9, pObs = 0.026, p-value = 0.319; **rotating-door task:** N=7, pObs = 0.016, p-value = 0.174).

S7 – cTADA results

Table S7.1. Results for continuous time of acquisition diffusion analysis (cTADA) for all the networks and tasks tested in a group of Barbary macaques (*Macaca sylvanus*) at Trentham Monkey Forest (UK).

Task	Network	Asocial		Social		ΔAICc	Support	LRT (p)	Approach	Rate of transmission	Rate of acquisition	ILVs	%ST	CI95%	ΔAICc for other rates of acquisition[1]
		AICc	Akaike	AICc	Akaike										
**Blue/yellow**	Grooming	784.81	0.80	787.57	0.20	-2.76	3.98	0<br>(1)	Add/***Multi***	***Con***/Non	Weibull	contactlevel,<br>freqacc	0%	s' = 0 (SE = 17.31)<br>Lower = 0<br>Upper = 36.79	Con: 1.18<br>Gamma: 0.02
	Huddling	784.81	0.80	787.57	0.20	-2.76	3.98	0<br>(1)	Add/***Multi***	***Con***/Non	Weibull	contactlevel,<br>freqacc	0%	s' = 0 (SE = 11.59)<br>Lower = 0<br>Upper = 18.21	Con: 5.84<br>Gamma: 3.19
	Proximity<br>1m	782.16	0.77	784.59	0.23	-2.43	3.37	0.539<br>(0.463)	Multi	Non	Weibull	socialrankclasshigh<br>contactlevel,<br>freqacc	13.0%	s' = 120.02 (SE = 221.22)<br>Lower = 0<br>Upper = 790.26	Con: 12.72<br>Gamma: 8.17
	Proximity<br>5m	782.95	0.49	782.90	0.51	0.05	1.03	3.249<br>(0.071)	Multi	Non	Weibull	socialrankclasshigh<br>contactlevel,<br>optionpref,<br>freqacc	52.5%	s' = 96.03 (SE = 157.92)<br>Lower = 0<br>Upper = 513.56	Con: 27.34<br>Gamma: 20.09
	Observation<br>1m	782.16	0.71	783.98	0.29	-1.82	2.48	1.151<br>(0.283)	Multi	Non	Weibull	socialrankclasshigh<br>contactlevel,<br>freqacc	13.1%	s' = 3.11 (SE = 3.94)<br>Lower = 0<br>Upper = 13.55	Con: 5.67<br>Gamma: 3.98
	Observation<br>5m	782.16	0.76	784.50	0.24	-2.34	3.23	0.626<br>(0.429)	Multi	Non	Weibull	socialrankclasshigh<br>contactlevel,<br>freqacc	8.1%	s' = 1.16 (SE = 1.78)<br>Lower = 0<br>Upper = 6.41	Con: 3.02<br>Gamma: 0.94
	Kinship	782.16	0.76	784.51	0.24	-2.35	3.24	0.617<br>(0.432)	Multi	Non	Weibull	socialrankclasshigh<br>contactlevel,<br>freqacc	8.6%	s' = 6.72 (SE = 10.63)<br>Lower = 0<br>Upper = 35.89	Con: 4.46<br>Gamma: 2.61
**Push/lift-up**	*Grooming*	*613.15*	*0.33*	*611.71*	*0.67*	*1.44*	*2.05*	*4.178<br>(0.041)*	*Multi*	*Non*	*Con*	*contactlatencynorm,<br>refillsobs*	*27.4%*	*s' = 1,146.04 (SE = 838.56)<br>Lower = 29.73<br>Upper = 3,733.45*	Gamma: 0.20<br>Weibull: -0.34
	Huddling	***613.15***	***0.05***	***607.40***	***0.95***	***5.75****	***17.69***	***8.485<br>(0.004)***	***Multi***	***Non***	***Con***	*contactlatencynorm,<br>refillsobs*	***44.1%***	***s' = 1,048.89 (SE = 667.06)<br>Lower = 200.64<br>Upper = 2,930.07***	Gamma: 4.06<br>Weibull: 3.09
	Proximity<br>1m	*613.15*	*0.32*	*611.63*	*0.68*	*1.52*	*2.14*	*4.258<br>(0.039)*	*Multi*	*Non*	*Con*	*contactlatencynorm,<br>refillsobs*	*36.9%*	*s' = 958.80 (SE = 747.39)<br>Lower = 28.18<br>Upper = 3,632.91*	Gamma: -0.04<br>Weibull: -0.82
	Proximity<br>5m	*613.15*	*0.42*	*612.51*	*0.58*	*0.63*	*1.37*	*3.374<br>(0.066)*	*Multi*	*Non*	*Con*	*contactlatencynorm,<br>refillsobs*	*43.9%*	*s' = 109.12 (SE = 101.90)<br>Lower = 0<br>Upper = 636.84*	Gamma: -1.06<br>Weibull: -1.99
	Observation<br>1m	***618.68***	***0.01***	***610.30***	***0.99***	***8.36****	***65.88***	***11.364<br>(0.001)***	***Multi***	***Con***	***Con***	***contactlatencynorm,<br>optionpref, freqacc***	***32.7%***	***s' = 2.06 (SE = 1.10)<br>Lower = 0.55***	Gamma: 13.67<br>Weibull: 7.57

														Upper = 5.33	
	Observation 5m	***618.68***	***0.00***	***602.89***	***1.00***	***15.78****	***2669.58***	***18.767 (0.000)***	***Multi***	***Non***	***Con***	***contactlatencynorm, optionpref, freqacc***	***73.5%***	***s' = 56.82 (SE = 40.28) Lower = 14.17 Upper = 267.89***	Gamma: 21.23 Weibull: 14.67
	Kinship	*613.15*	*0.37*	*612.05*	*0.63*	*1.09*	*1.73*	*3.836 (0.050)*	*Multi*	*Non*	*Con*	*contactlatencynorm, refillsobs*	*23.9%*	*s' = 35.79 (SE = 27.12) Lower = 0 Upper = 77.70*	Gamma: 0.16 Weibull: -0.20
**Rotating-door**	*Grooming*	*334.21*	*0.40*	*333.36*	*0.60*	*0.85*	*1.53*	*3.928 (0.047)*	*Multi*	*Con*	*Con*	*contactlevel*	*33.98 %*	*s' = 122.44 (SE = 100.59) Lower = 0.76 Upper = 486.77*	Gamma: -0.16 Weibull: -0.49
	Huddling	332.97	0.76	335.26	0.24	-2.29	3.13	1.351 (0.245)	Multi	Con	Con	contactlevel, optionpref	28.9%	s' = 24.69 (SE = 36.63) Lower = 0 Upper = 210.58	Gamma: -4.14 Weibull: -4.20
	Proximity 1m	326.32	0.61	327.24	0.39	-0.91	1.58	2.162 (0.141)	Multi	Non	Con	contactlatencynorm	36.7%	s' = 1,729.01 (SE = 5722.40) Lower = 0 Upper = 9,095.52	Gamma: -2.10 Weibull: -2.53
	Proximity 5m	326.32	0.67	327.76	0.33	-1.44	2.05	1.637 (0.201)	Multi	Non	Con	contactlatencynorm	38.6%	s' = 199.93 (SE = 240.14) Lower = 0 Upper = 1,451.89	Gamma: -2.66 Weibull: -3.11
	Observation 1m	***334.21***	***0.07***	***328.95***	***0.93***	***5.25****	***13.84***	***8.331 (0.004)***	***Multi***	***Non***	***Con***	***contactlevel***	***12.8%***	***s' = 351.31 (SE = 300.54) Lower = 38.11 Upper = 1,601.04***	Gamma: 5.48 Weibull: 6.26
	Observation 5m	***332.97***	***0.08***	***328.06***	***0.92***	***4.91****	***11.66***	***8.549 (0.003)***	***Multi***	***Con***	***Con***	***contactlevel, optionpref***	***50.4%***	***s' = 2.43 (SE = 1.69) Lower = 0.41 Upper = 8.91***	Gamma: 2.65 Weibull: 1.99
	Kinship	*332.97*	*0.38*	*332.00*	*0.62*	*0.97*	*1.62*	*4.603 (0.032)*	*Multi*	*Non*	*Con*	*contactlevel, optionpref*	*27.4%*	*s' = 132.53 (SE = 107.57) Lower = 7.26 Upper = 510.31*	Gamma: -1.20 Weibull: -1.39

Asocial: Purely asocial learning model. **Social:** Asocial + social learning model. **ΔAICc:** Difference in AIC between asocial and asocial+social learning models. **Support:** The degree to which the agent-based model (asocial or social) with the lowest AIC is better than the alternative. For example, in the first line the asocial learning model for grooming was 4.41x better than the corresponding asocial + social learning model for this network. **Approach:** Additive (Add), Multiplicative (Multi) or Unconstrained (Unc) model. **Rate of transmission:** Constant (Con), Non-constant (Non). When models provided the same results using different approaches and rates, LRT and CI95% were calculated for those with better estimates of the s' parameter (underlined). * indicates models that provide evidence of social transmission according to ΔAICc (bold and italics (dark grey shading) = enough evidence; only italics (light grey shading) = almost enough evidence).. For interpretation of CI95% for the *s'* parameter, refer to Table 1 and SI in Hasenjager et al. (2020) [36]. [1] Results of ΔAICc using other baseline rates of acquisition maintaining the approach and rate of transmission of the best model.

Due to collinearity, social rank order, contact latency and preferred option were removed from the analyses (OADA and cTADA) for blue/yellow task, and contact level and social rank order were not included in the NBDA for push/lift-up and rotating-door tasks. Since contact level and social rank order are variables that measure similar attributes as contact latency and social rank class, respectively, they were used instead when errors of convergence in the optimization algorithm persisted even after the optimization method used in the regression model was changed.

By default, NBDA assumes that all individuals perform the target behaviour at a similar rate (constant rate of social transmission). The variable 'rate of performance with the task or social transmission of the trait' (***Table 5*** in Main Text) was calculated and included in the NBDA to test for the influence on social diffusion of non-constant rates of social transmission. Regarding asocial acquisition, a constant baseline function considers that there are multiple steps individuals must undergo to learn a novel task, but the time taken to solve each step is unequal. Therefore, the distribution of times to solve (latencies) asocially tends to an exponential distribution. If the average solving time for each step is considered equal, the latencies follow a gamma distribution (non-constant baseline rate). The Weibull distribution is a continuous probability distribution that can fit an extensive range of distribution shapes. Therefore, it is commonly used as a flexible non-constant baseline function. For more information on these functions, see supplementary information in Hasenjager et al. (2021) [36].

For those cTADA models that provided evidence of social transmission using the social networks informed, comparisons with a homogeneous network were conducted to determine whether social transmission followed the provided social network [36]. Results can be found in ***Table S7.2***.

Table S7.2. Comparisons of cTADA models that provided evidence of social transmission in a group of Barbary macaques (*Macaca sylvanus*) at Trentham Monkey Forest (UK) using social network data vs cTADA using homogenous networks.

Task	Homogenous network	Social network	ΔAICc	Support
**Push/lift-up task**	s' = 2.48 (SE = 2.36)<br>ILVs = contactlatencynorm, refillsobs<br>AICc = 611.06	Grooming (AICc = 611.71)	-0.65	1.38
		Huddling (AICc = 607.40)	3.66	6.24*
		Proximity 1m (AICc = 611.63)	-0.57	1.33
		Proximity 5m (AICc = 612.51)	-1.45	2.06
		Observation 1m (AICc = 610.30)	0.76	81.58*
		Observation 5m (AICc = 602.89)	8.17	2842.39*
		Kinship (AICc = 612.05)	-0.99	1.64
**Rotating-door task**	s' = 0.15 (SE = 0.21)<br>ILVs = contactlevel<br>AICc = 335.73	Grooming (AICc = 333.36)	2.37	3.27*
		Observation 1m (AICc = 328.95)	6.78	21.66*
		Observation 5m (AICc = 328.06)	7.67	16.96*
		Kinship (AICc = 332.00)	3.73	1.57*

ΔAICc: Difference in AIC between the asocial + social learning models using each social network vs the corresponding homogeneous network. **Support:** The degree to which the agent-based model (obtained using social or homogeneous network) with the lowest AIC is better than the alternative. For example, in the first line, the asocial + social learning model for the homogeneous network is 1.38x better than the asocial + social learning model for grooming. * indicates those models for which the social network provides more support to social transmission than the homogeneous network.

Similar conclusions could be drawn from both OADA and cTADA for the blue/yellow task except in the case of 5m proximity network, where the asocial + social learning model was slightly better than the asocial learning model. However, evidence of social transmission was not sufficient in any case for the blue/yellow task (***Table 6*** in ***Main Text*** and ***Table S7.1***).

For the push/lift-up task, all best cTADA models indicated that the asocial + social learning model explained the data better than the corresponding asocial learning model, as opposed to OADA, where this was only found for huddling and both observation networks. According to ΔAICc, only huddling and observation networks provided sufficient (strong in this case: ΔAICc > 4) evidence of social transmission (***Table 6*** in ***Main Text*** and ***Table S7.1***). However, in all cases, except for the 5m proximity network, LRT results suggest evidence of an effect consistent with social transmission ($p < 0.05$).

cTADA results for the rotating-door task showed that the asocial + social learning model was better than the asocial learning model in grooming, kinship and observation networks, as opposed to OADA, where this only was found for observation networks. This was also reflected in the significant p values of the LRT for these networks. However, sufficient evidence of social transmission (ΔAICc > 2) was only found in cTADA models informed with observation networks, as in OADA (***Table 6*** in ***Main Text*** and ***Table S7.1***).

Only huddling and observation networks in the push/lift-up task provided more support to social transmission than homogenous networks in cTADA. All networks that provided evidence of social transmission in cTADA for the rotating-door task explained the data better than a homogenous network, indicating that the transmission pathways likely followed the social network provided [36].

Using a different baseline rate of acquisition for the best cTADA models (i.e., maintaining the approach and rate of transmission of the best model) provided, in most cases, opposite results in terms of which agent-based model (asocial or asocial + social) better explained the data. The only exceptions were 5m proximity networks in the blue/yellow task, huddling and both observation networks in the push/lift-up and rotating-door tasks, and both proximity networks in the rotating-door task (***Table S7.1***). When different baseline rates of acquisition were tested along with other combinations of approach and rate of transmission, only huddling and 1m observation networks in the push/lift-up task, and huddling and both proximity networks in the rotating-door task resulted in the same best agent-based model. This

suggests that the analysis is dominated by the time course of events as opposed to the pattern of diffusion through the network. In these cases, results of OADA are preferable to those of TADA [36].

S8 – Further discussion on social learning

Hoppitt (2017) empirically demonstrated that observation networks are a direct and powerful way to detect social transmission, even when there is no social structure information or when other networks (e.g. affiliative) cannot provide evidence of social learning [37]. This is because NBDA can provide evidence for social learning if the order in which individuals observe each other follows the order of diffusion [37]. Our results support this statement since networks based on who observed whom during task introductions provided the strongest evidence of social learning.

The effect of number of refills on accelerating learning rates may be due to an increased motivation to interact with the task of those observing rewards being placed inside of it. The slightly faster learning rates for push and counter-clockwise options compared to their alternatives may be due to the fact that lift-up and clockwise actions apparently required the use of, at least, two body parts to retrieve rewards: one to open and hold the door, and one to reach out for raisins. Individuals generally used the same hand to push or move the door counter-clockwise, hold it and retrieve the raisins.

Evidence of social transmission was obtained using the multiplicative approach in all cases, which assumes that a social influence multiplies the chances to learn asocially. Accordingly, social transmission in our group of Barbary macaques likely occurred through indirect social learning processes [38] whereby attention is drawn to the task socially but individuals learn how to solve it for themselves. The fact that *frequency of access* influenced social transmission in those within 1m (who require higher levels of social tolerance than those at 5m) and that individuals did not seem to copy the actions observed in the push/lift up task, suggests that macaques learned this task asocially motivated by the presence of a demonstrator at task (stimulus/local enhancement or social facilitation) [38]. Accordingly, individuals could have been attracted to the task and/or increased their rate of task exploration due to the mere presence of another individual interacting with it [39]. Previous studies have also found evidence of stimulus/local enhancement and social facilitation in macaque species (lion-tailed macaques, *Macaca silenus*, [40]; long-tailed macaques, *Macaca fascicularis*, [41][42]; Japanese macaques, *Macaca fuscata*, [43]) and other primate species [44][45][46][47][48].

Close-proximity observations (1m), which led to the strongest evidence of social learning in the most difficult task (rotating-door), allow the transmission of detailed information. The fact that individuals copied the preferred action of the most successful individual observed in the rotating-door task suggests that a transient effect of the action observed might have led Barbary macaques to perform

the same successful actions observed [39]. Accordingly, *frequency of access to the task* might have had no effect on social transmission if individuals had not observed the actions being used and had not had immediate access to the task. We suggest that social learning of the rotating-door task occurred via response facilitation. Here, individuals perform a motor action already in the species behavioural repertoire, or use a familiar action in a novel context [39]. Barbary macaques used the rotating door by pushing it aside (i.e. making the door rotate clockwise or counter-clockwise around a screw; novel context). Pushing is a motor action already in the behavioural repertoire of Barbary macaques [49]. Evidence for response facilitation has also been found in macaques (pig-tailed macaques, *Macaca nemestrina*, [50]; long-tailed macaques, [51]) and other primate species (great apes, [52]). Response facilitation seems unlikely for the push/lift-up task, as there was no evidence that individuals performed the same rewarding actions observed [38].

Our results have similarities to findings with wild monkeys. In a task similar to our push/lift-up task, there was evidence for indirect social learning (i.e. stimulus and local enhancement) in wild vervets [46], as in the present study. However, the results presented in our study must be taken with caution since data were only collected for a short period in one group of Barbary macaques.

REFERENCES:

[1] Funkhouser, J. A., Mayhew, J. A., Sheeran, L. K., Mulcahy, J. B., & Li, J. H. (2018). Comparative investigations of social context-dependent dominance in captive chimpanzees (*Pan troglodytes*) and wild Tibetan macaques (*Macaca thibetana*). *Scientific reports*, *8*(1), 1-15. https://doi.org/10.1038/s41598-018-32243-2

[2] Drews, C. (1993). The concept and definition of dominance in animal behaviour. *Behaviour*, *125*(3-4), 283-313. https://doi.org/10.1163/156853993X00290

[3] Vessey, S. H. (1981). Dominance as control. *Behavioral and Brain Sciences*, *4*(3), 449-449. https://doi.org/10.1017/S0140525X00009845

[4] Albers, P. C., & de Vries, H. (2001). Elo-rating as a tool in the sequential estimation of dominance strengths. *Animal Behaviour*, 489-495. https://doi.org/10.1006/anbe.2000.1571

[5] Douglas, P. H., Ngomo, A. C. N., & Hohmann, G. (2017). A novel approach for dominance assessment in gregarious species: ADAGIO. *Animal Behaviour*, *123*, 21-32. https://doi.org/10.1016/j.anbehav.2016.10.014

[6] Fushing, H., McAssey, M. P., Beisner, B., & McCowan, B. (2011). Ranking network of a captive rhesus macaque society: a sophisticated corporative kingdom. *PloS one*, *6*(3), e17817. https://doi.org/10.1371/journal.pone.0017817

[7] de Vries, H. (1995). An improved test of linearity in dominance hierarchies containing unknown or tied relationships. *Animal Behaviour*, *50*(5), 1375-1389. https://doi.org/10.1016/0003-3472(95)80053-0

[8] de Vries, H. (1998). Finding a dominance order most consistent with a linear hierarchy: a new procedure and review. *Animal Behaviour*, *55*(4), 827-843. https://doi.org/10.1006/anbe.1997.0708

[9] Schmid, V. S., & de Vries, H. (2013). Finding a dominance order most consistent with a linear hierarchy: an improved algorithm for the I&SI method. *Animal Behaviour*, *86*(5), 1097-1105. https://doi.org/10.1016/j.anbehav.2013.08.019

[10] Leiva, D., Solanas, A., de Vries, H., & Kenny, D. A. (2010, August). DyaDA: an R package for dyadic data analysis. In *Proceedings of measuring behavior* (Vol. 2010), 162-165.

[11] David, H. A. (1987). Ranking from unbalanced paired-comparison data. *Biometrika*, *74*(2), 432-436. https://doi.org/10.1093/biomet/74.2.432

[12] Gammell, M. P., de Vries, H., Jennings, D. J., Carlin, C. M., & Hayden, T. J. (2003). David's score: a more appropriate dominance ranking method than Clutton-Brock et al.'s index. *Animal behaviour*, *66*(3), 601-605. https://doi.org/10.1006/anbe.2003.2226

[13] de Vries, H., Stevens, J. M., & Vervaecke, H. (2006). Measuring and testing the steepness of dominance hierarchies. *Animal Behaviour*, *71*(3), 585-592. https://doi.org/10.1016/j.anbehav.2005.05.015

[14] de Vries, H. (2009). On using the DomWorld model to evaluate dominance ranking methods. *Behaviour*, *146*(6), 843-869. https://doi.org/10.1163/156853909X412241

[15] Neumann, C. *et al.* A. (2011). Assessing dominance hierarchies: validation and advantages of progressive evaluation with Elo-rating. *Animal Behaviour*, *82*(4), 911-921. https://doi.org/10.1016/j.anbehav.2011.07.016

[16] van Hooff, J. A., & Wensing, J. A. (1987). Dominance and its behavioral measures in a captive wolf pack. *Man and wolf: Advances, issues, and problems in captive wolf research*, *4*, 219.

[17] van Schaik, C. P. (1989). The ecology of social relationships amongst female primates. Comparative socioecology: The behavioral ecology of humans and other mammals, 195-218. https://ci.nii.ac.jp/naid/20000904492/

[18] Thierry, B. (2007). Unity in diversity: lessons from macaque societies. *Evolutionary Anthropology: Issues, News, and Reviews: Issues, News, and Reviews*, *16*(6), 224-238. https://doi.org/10.1002/evan.20147

[19] Jenks, G. F. (1967). The data model concept in statistical mapping. *International yearbook of cartography*, *7*, 186-190. NAID: 10021899676

[20] Foerster, S. *et al.* (2016). Chimpanzee females queue but males compete for social status. *Scientific reports*, *6*(1), 1-11. https://doi.org/10.1038/srep35404

[21] Bray, J., & Gilby, I. C. (2020). Social relationships among adult male chimpanzees (*Pan troglodytes schweinfurthii*): variation in the strength and quality of social bonds. *Behavioral Ecology and Sociobiology*, *74*(9), 1-19. https://doi.org/10.1007/s00265-020-02892-3

[22] Bray, J. R. D. (2021). *Social Relationships in Male Chimpanzees: Form, Function, and Development* (Doctoral dissertation). Arizona State University, USA. Retrieved from https://keep.lib.asu.edu/items/161298

[23] Feldblum, J., Foerster, S., Franz, M. (2021). EloOptimized: optimized Elorating method for obtaining dominance ranks, https://CRAN.Rproject.org/package=EloOptimized

[24] Kendal, R. L., Coe, R. L., & Laland, K. N. (2005). Age differences in neophilia, exploration, and innovation in family groups of callitrichid monkeys. *American Journal of Primatology: Official Journal of the American Society of Primatologists*, *66*(2), 167-188. https://doi.org/10.1002/ajp.20136

[25] Jacobs, G. H. (1996). Primate photopigments and primate color vision. *Proceedings of the National Academy of Sciences*, *93*(2), 577-581. https://doi.org/10.1073/pnas.93.2.577

[26] Onishi, A. *et al.* (1999). Dichromatism in macaque monkeys. *Nature*, *402*(6758), 139-140. https://doi.org/10.1038/45966

[27] Carvalho, L. S., Pessoa, D., Mountford, J. K., Davies, W. I., & Hunt, D. M. (2017). The genetic and evolutionary drives behind primate color vision. *Frontiers in ecology and Evolution*, *5*, 34. https://doi.org/10.3389/fevo.2017.00034

[28] Skalnikova, P., Frynta, D., Abramjan, A., Rokyta, R., & Nekovářová, T. (2020). Spontaneous color preferences in rhesus monkeys: What is the advantage of primate trichromacy? *Behavioural processes*, *174*, 104084. https://doi.org/10.1016/j.beproc.2020.104084

[29] Mollon, J. D. (1989). "Tho' she kneel'd in that place where they grew…" The uses and origins of primate colour vision. *Journal of Experimental Biology*, *146*(1), 21-38. PMID 2689563.

[30] Bowmaker, J. K., Astell, S., Hunt, D. M., & Mollon, J. D. (1991). Photosensitive and photostable pigments in the retinae of Old World monkeys. *Journal of experimental Biology*, *156*(1), 1-19.

[31] Kendal, R. L. *et al.* (2010). Evidence for social learning in wild lemurs (*Lemur catta*). *Learning & Behavior, 38*(3), 220–234. http://doi.org/10.3758/LB.38.3.220

[32] Fleiss, J. L., Levin, B., & Paik, M. C. (2003). Statistical methods for rates and proportions (3rd ed.). Hoboken, NJ: Wiley.

[33] McHugh, M. L. (2012). Interrater reliability: the kappa statistic. *Biochemia medica: Biochemia medica*, *22*(3), 276-282. https://hrcak.srce.hr/89395

[34] Maclure, M., & Willett, W. C. (1987). Misinterpretation and misuse of the kappa statistic. *American journal of epidemiology*, *126*(2), 161-169. https://doi.org/10.1093/aje/126.2.161

[35] Kendal, R. L., Kendal, J. R., Hoppitt, W., & Laland, K. N. (2009). Identifying social learning in animal populations: a new 'option-bias' method. *PLoS One, 4*(8), e6541. https://doi.org/10.1371/journal.pone.0006541

[36] Hasenjager, M. J., Leadbeater, E., & Hoppitt, W. (2021). Detecting and quantifying social transmission using network-based diffusion analysis. *Journal of Animal Ecology*, *90*(1), 8-26. https://doi.org/10.1111/1365-2656.13307

[37] Hoppitt, W. (2017). The conceptual foundations of network-based diffusion analysis: choosing networks and interpreting results. *Philosophical Transactions of the Royal Society B: Biological Sciences*, *372*(1735), 20160418. https://doi.org/10.1098/rstb.2016.0418

[38] Hoppitt, W., & Laland, K. N. (2011). Detecting social learning using networks: a users guide. *American Journal of Primatology*, *73*(8), 834-844. https://doi.org/10.1002/ajp.20920

[39] Hoppitt, W., & Laland, K. N. (2008). Social processes influencing learning in animals: a review of the evidence. *Advances in the Study of Behavior, 38*, 105–165. http://doi.org/10.1016/S0065-3454(08)00003-X

[40] Westergaard, G. C., & Lindquist, T. (1987). Manipulation of objects in a captive group of lion-tailed macaques (*Macaca silenus*). *American journal of primatology*, *12*(2), 231-234. https://doi.org/10.1002/ajp.1350120211

[41] Zuberbühler, K., Gygax, L., Harley, N., & Kummer, H. (1996). Stimulus enhancement and spread of a spontaneous tool use in a colony of long-tailed macaques. *Primates*, *37*(1), 1-12. https://doi.org/10.1007/BF02382915

[42] Schmitt, V., Schloegl, C., & Fischer, J. (2014). Seeing the experimenter influences the response to pointing cues in long-tailed macaques. *PloS one*, *9*(3), e91348. https://doi.org/10.1371/journal.pone.0091348

[43] Leca, J. B., Gunst, N., & Huffman, M. A. (2010). The first case of dental flossing by a Japanese macaque (*Macaca fuscata*): implications for the determinants of behavioral innovation and the constraints on social transmission. *Primates*, *51*(1), 13. https://doi.org/10.1007/s10329-009-0159-9

[44] Custance, D., Whiten, A., Sambrook, T., & Galdikas, B. (2001). Testing for social learning in the" artificial fruit" processing of wildborn orangutans (*Pongo pygmaeus*), Tanjung Puting, Indonesia. *Animal Cognition*, *4*(3), 305-313. https://doi.org/10.1007/s100710100100

[45] Hook, M. A., Lambeth, S. P., Perlman, J. E., Stavisky, R., Bloomsmith, M. A., & Schapiro, S. J. (2002). Inter-group variation in abnormal behavior in chimpanzees (*Pan troglodytes*) and rhesus macaques (*Macaca mulatta*). *Applied Animal Behaviour Science*, *76*(2), 165-176. https://doi.org/10.1016/S0168-1591(02)00005-9

[46] van de Waal, E., Renevey, N., Favre, C. M., & Bshary, R. (2010). Selective attention to philopatric models causes directed social learning in wild vervet monkeys. *Proceedings of the Royal Society B: Biological Sciences*, *277*(1691), 2105-2111. https://doi.org/10.1098/rspb.2009.2260

[47] Kendal, R. *et al.* (2015). Chimpanzees copy dominant and knowledgeable individuals: implications for cultural diversity. Evolution and Human Behavior, 36(1), 65–72. http://doi.org/10.1016/j.evolhumbehav.2014.09.002

[48] Bandini, E., & Tennie, C. (2020). Exploring the role of individual learning in animal tool-use. *PeerJ*, *8*, e9877. https://doi.org/10.7717/peerj.9877

[49] Amici, F., Caicoya, A. L., Majolo, B., & Widdig, A. (2020). Innovation in wild Barbary macaques (*Macaca sylvanus*). *Scientific Reports*, *10*(1), 1-12. https://doi.org/10.1038/s41598-020-61558-2

[50] Ferrari, P. F., Maiolini, C., Addessi, E., Fogassi, L., & Visalberghi, E. (2005). The observation and

hearing of eating actions activates motor programs related to eating in macaque monkeys. *Behavioural brain research*, *161*(1), 95-101. https://doi.org/10.1016/j.bbr.2005.01.009

[51] Brotcorne, F. *et al.* (2020). Social influence on the expression of robbing and bartering behaviours in Balinese long-tailed macaques. Animal cognition, 23(2), 311-326. https://doi.org/10.1007/s10071-019-01335-5

[52] Amici, F., Aureli, F., & Call, J. (2014). Response facilitation in the four great apes: is there a role for empathy? *Primates*, *55*(1), 113-118. https://doi.org/10.1007/s10329-013-0375-1
